# Robust and Biodegradable Heterogeneous Electronics with Customizable Cylindrical Architecture for Interference-Free Respiratory Rate Monitoring

**DOI:** 10.1007/s40820-025-01879-x

**Published:** 2025-08-19

**Authors:** Jing Zhang, Wenqi Wang, Sanwei Hao, Hongnan Zhu, Chao Wang, Zhouyang Hu, Yaru Yu, Fangqing Wang, Peng Fu, Changyou Shao, Jun Yang, Hailin Cong

**Affiliations:** 1 School of Materials Science and Engineering, Shandong University of Technology, Zibo, 255000 People’s Republic of China; 2 Beijing Key Laboratory of Lignocellulosic Chemistry, College of Materials Science and Technology, Beijing Forestry University, Beijing, 100083 People’s Republic of China; 3 Liaoning Key Laboratory of Lignocellulose Chemistry and Biomaterials, College of Light Industry and Chemical Engineering, Dalian Polytechnic University, Dalian, 116034 People’s Republic of China; 4 School of Agricultural Engineering and Food Science, Shandong University of Technology, Zibo, 255000 People’s Republic of China

**Keywords:** Wearable electronics, Piezoresistive sensor, Heterogeneous, Cellulose, Respiratory monitoring

## Abstract

A rapidly growing field is piezoresistive sensor for accurate respiration rate monitoring to suppress the worldwide respiratory illness. However, a large neglected issue is the sensing durability and accuracy without interference since the expiratory pressure always coupled with external humidity and temperature variations, as well as mechanical motion artifacts. Herein, a robust and biodegradable piezoresistive sensor is reported that consists of heterogeneous MXene/cellulose-gelation sensing layer and Ag-based interdigital electrode, featuring customizable cylindrical interface arrangement and compact hierarchical laminated architecture for collectively regulating the piezoresistive response and mechanical robustness, thereby realizing the long-term breath-induced pressure detection. Notably, molecular dynamics simulations reveal the frequent angle inversion and reorientation of MXene/cellulose in vacuum filtration, driven by shear forces and interfacial interactions, which facilitate the establishment of hydrogen bonds and optimize the architecture design in sensing layer. The resultant sensor delivers unprecedented collection features of superior stability for off-axis deformation (0–120°, ~ 2.8 × 10^–3^ A) and sensing accuracy without crosstalk (humidity 50%–100% and temperature 30–80 °C). Besides, the sensor-embedded mask together with machine learning models is achieved to train and classify the respiration status for volunteers with different ages (average prediction accuracy ~ 90%). It is envisioned that the customizable architecture design and sensor paradigm will shed light on the advanced stability of sustainable electronics and pave the way for the commercial application in respiratory monitory.

**Supplementary Information:**

The online version contains supplementary material available at 10.1007/s40820-025-01879-x.

## Highlights

- Piezoresistive sensor in tandem with customizable cylindrical microstructure for ultra-sensitive, stable, and interference-free performance.
- Molecular dynamics simulations reveal shear-force-driven self-assembly mechanisms.
- Eco-friendly and robust sensing layer for scalable, sustainable fabrication.

**Supplementary Information:**

The online version contains supplementary material available at 10.1007/s40820-025-01879-x.

## Graphical abstract

**Figure:**
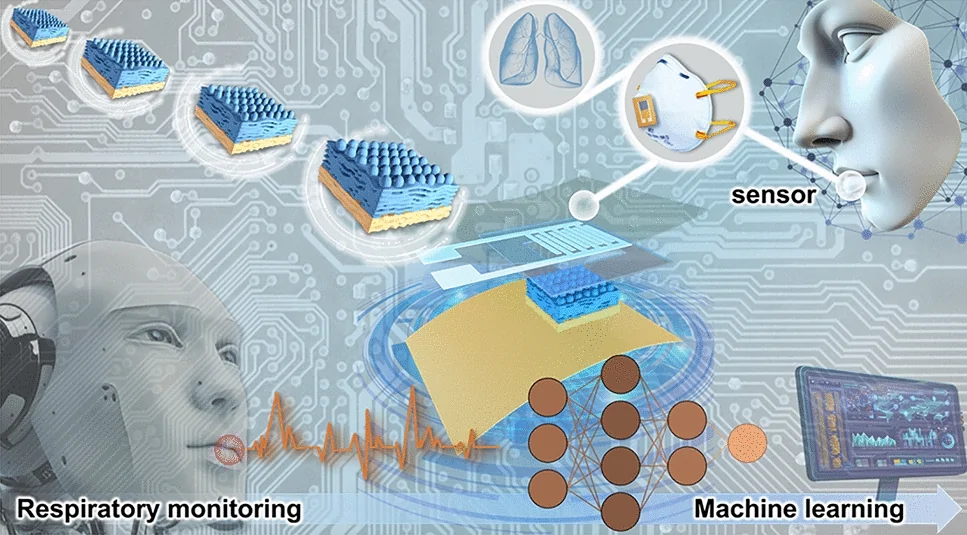

## Introduction

Respiration rate (RRT), defined as breaths per minute, holds a pivotal role in clinical evaluation because of the superior discriminatory capability in comparison with blood pressure and pulse rate, achieving the discernment of high-risk patient cohorts in the context of cardio-pulmonary catastrophic deterioration. From a clinical context, the measurement of RRT includes spirometry, capnometry, and pneumography, while these techniques usually involve complex and bulky apparatus, potentially disrupting the natural breathing process [1–4]. In continuous monitoring scenarios, such as dynamic assessment, rehabilitation training, and sleep studies, the use of the above complex devices can be extremely limited. Thus, there is an urgent need to develop wearable respiration monitoring electronics to assist clinical monitoring [5–7]. Up to now, according to various sensing mechanisms, the piezoresistive sensor features a simple structure, outstanding sensitivity, and fast response time [8–10], which is currently the most promising candidate for respiration monitoring since expiratory intensity can greatly cause piezoresistive effect to achieve RRT measurement. However, to satisfy the accurate acquisition in multiple respiratory states, a huge challenge is to achieve supersensitivity without signal distortion and maintain structural integrity since mechanical mismatching, stress concentration, and crack propagation during monitoring can largely affect the signal accuracy and reduce the sensing stability [11–13]. Furthermore, in real-world applications, wearable sensors are frequently subjected to complex mechanical deformations, such as bending, stretching, and twisting as well as environmental fluctuations in humidity and temperature. These factors can interact with the sensor materials, potentially inducing microstructural changes, affecting interfacial bonding, and influencing the piezoresistive response. Therefore, it is essential to design sensor architectures and select materials that can withstand such combined stresses while maintaining high sensing accuracy and stability. Besides, with the increasing demand for electronic products, the huge discarded toxic substances and electronic wastes cause serious environmental concerns and resource depletion [14]. Therefore, an imperative arises is to explore sustainable materials integrated wearable electronics achieving sensitive RRT monitoring without signal distortion for facilitating continuous health surveillance.

Currently, considerable efforts have been devoted to achieve the combination of raw materials selection and structural design for natural material integrated piezoresistive sensors, which admirably overcome the obstacle of electronic wastes and gain the desirable electrochemical performances [15–19]. For the former, the careful selection of appropriate substrate and active materials (e.g., cellulose, gelatin, and chitosan) is pivotal for achieving collection characteristics for RRT monitoring sensors, including exceptional robustness, sustainability, biocompatibility, and widespread availability [20–22]. For the latter, various device structures have been proposed for sensitive piezoresistive sensors via including multilayer designs, three‐dimensional architectures (e.g., pyramid, prism, and dome), hierarchical layouts, and so forth, realizing the optimization of structural compressibility and the regulation of piezoresistive effect upon sensing–electrode interface, thereby solving the unreconciled trade-off between sensing sensitivity and durability. For example, He et al. [23] designed an intrinsically stretchable and patternable tactile sensor array with conformal wrinkled graphene structure enabling strain insensitivity, maintaining stable performances in long strain range (0%–100%). Alternatively, Chen and co-workers [10] proposed a kirigami-inspired pressure sensor based on polydimethylsiloxane (PDMS)/Ti_3_C_2_T_x_ MXene films, exhibiting strain-invariant conductivity, high sensitivity (66.3 nF kPa^−1^), and cycle stability over 1000 cycles that provided a creative way for solving strain interference. Despite the impressive results, the implementation of piezoresistive sensor for RTT measurement is still largely frustrated to the following challenges: (1) The exhaled gas-mediated resistance variations of sensor also combine with multi-stimulus signals that involved off-axis deformations (e.g., bending, twisting, and wrinkling), humidity accumulation, and heat diffusion, which will inevitably weak structural stability and can be leveraged to engender an electric signal on sensing components, especially under the continuous and dynamic breathing conditions [24, 25]. (2) The complexity of structural engineering involves expensive fabrication and complicated techniques (photolithography, pattern growth, laser, screen or ink-jet printing, impregnation, and sputtering) to patterning the sensing layer, thus greatly impeding the scalability and customizability for streamlined fabrication and wide application scope [26]. More importantly, however, a few studies have widely reported some efficient engineering techniques (laser, vacuum filtration, and hot-pressing) to verify the structure effectiveness for optimizing materials properties [27–31]. However, how to expand technology advancement and further elaborate the underlying construction mechanism, understanding structurally cooperative mechanical effects, is still rarely reported. Consequently, it is of paramount importance to overcome the undesirable signal distortion and solve the structural vulnerability through a facile fabrication technique and further interpret the mechanism, engineering a sustainable and ultra-sensitive piezoresistive sensor paradigm toward dynamic respiratory monitoring.

Herein, we propose a heterogeneous engineering strategy in combination with cylindrical microstructure design and green material integration, contributing to the construction of ultra-sensitive and stable sensor paradigm for respiratory monitoring without signal distortion. The cellulose-based piezoresistive sensor (CPS) is facilely fabricated that mainly relies on template-assisted vacuum filtration method for constructing a heterogeneous sensing layer consisting of an MXene/cellulose nanofibrils conductive layer and a gelatin-based robust layer, together with screen-printing technique for Ag interdigital electrode. As the most striking highlight, this compact heterogeneous design coupling with a cylindrical morphology collectively promotes the mechanical integrity and ultra-sensitive response for high-fidelity signal detection. We demystify the mechanism behind the architecture design via molecular dynamics (MD) simulations, which confirms that shear and filtration forces drive the alignment and stacking of MXene and cellulose, forming a compact, well-ordered conductive layer. Stabilized by hydrogen bonding, these interactions dissipate energy during deformation and enhance mechanical robustness. Also, on account of the sensitive response on customizable cylindrical morphology and the mechanical toughening via abundant interfacial bridges between conductive layer and robust layer, the proposed CPS delivered collection merits including outstanding bending resistance, biodegradability, and superior sensitivity, together with stable responsiveness upon multiple signal inputs including bending, humidity, and temperature variation, satisfying the complex breathing monitoring conditions. As a proof-of-concept, the substantial progress of accurate and stable RRT measurement in dynamics is realized without obvious signal superposition or fluctuation. The collected data are further conducted by machine learning algorithms for real-time breath evaluation, including normal, fast, deep, and cough patterns. This work possesses insightful significance to configure conveniently the individual products with various sensitive requirement for respiration rate discrimination without signal distortion and contributes to the advancement of sustainable electronics.

## Experimental Section

### Materials

The 400 mesh MAX phase (Ti_3_AlC_2_), hydrochloric acid (HCl, 36%), gelatin, and lithium fluoride (LiF, 99.7%) were obtained from Maclean Reagents Co. LTD. TEMPO-oxidized cellulose nanofiber (TOCNF) suspensions (~ 5.0 wt%) were sourced from Tianjin University of Science and Technology. The bacterial cellulose was gained from Qihong Technology Co., LTD. Silver (Ag) pastes were purchased from Jing Zhe Technology Co., LTD. All the above reagents were directly used without further purification.

### Fabrication of Delaminated MXenes

Delaminated Ti_3_C_2_T_x_ (MXene) was fabricated by etching the Ti_3_AlC_2_ MAX phase with LiF/HCl solution. Initially, 1 g Ti_3_AlC_2_ powder was slowly added into the mixed solution of LiF (1 g) and 20 mL HCl (9 M) in a high-density polyethylene bottle under stirring to eliminate the Al layers (35 °C, 24 h). The obtained etched samples are repeatedly centrifuged at 6000 r min^−1^ and washed in deionized water to remove residual acid and reaction by-products until the pH exceeded 6, yielding dark green supernatants. The resulting Ti_3_C_2_T_x_ concentration in the supernatant was approximately 2 mg mL^−1^. Then, the solution was centrifuged (3200 r min^−1^, 20 min) after ultrasonic treatment in an ice bath (1 h, Ar atmosphere). Following collection, the suspension was freeze-dried to yield MXene nanosheet powder and stored at 4 °C until further use.

### Preparation of Cylindrical Micro-Structure-Based Sensing Layer

TOCNF aqueous used in this work is obtained by diluting high-concentration colloid (5 wt%) and mixed with MXene nanosheet to gain a uniform suspension (totally 50 g). Then the self-assembly process was achieved via filtered on a microfiltration membrane with 0.65 μm pore size (diameter 5 cm) to form the wet cakes. To customize the cylindrical microstructure, a CO_2_ laser printer was used to create mask-defined stencils with varying aperture sizes (100, 200, 300, and 500 μm), enabling precise control over the structural features. Next, the residual suspension (20 g) was uniformly deposited across the surface of the wet cakes with a customizable template to form cylindrical microstructure via vacuum-assisted filtration. Finally, the above wet cakes were vacuum-dried at 60 °C for 12 h to fabricate the sensing layer.

### Preparation of Robust Heterogeneous Sensing Layer

A gelatin-based suppression layer with a thickness of approximately 1 mm was uniformly coated onto the substrate. The formulation of the suppression layer consisted of 5 g of gelatin, 10 g of deionized water, and 2.5 g of glycerol, corresponding to a gelatin concentration of 25 wt% relative to the total mass of the mixture. The components were thoroughly mixed and heated at 60 °C under constant stirring until a homogeneous solution was obtained. This mixture was then cast and allowed to cool under ambient conditions to form a stable, flexible gelatin-based film suitable for subsequent sensing layer assembly.

### Fabrication of Cellulose-Based Piezoresistive Sensor

The BC film as encapsulation layer was cut into a rectangle (20 mm × 70 mm × 2 mm) and placed on a horizontal platform. Ag-based interdigital electrode achieved the ohmic connection via designed screen-printed patterns (400 mesh). Specifically, the silver paste (5 g) was uniformly scraped off with a razor blade (50 mm min^−1^) to print the designed electrode and then heated at 30 °C for 20 min to solidify the Ag traces. A schematic of the electrode layout is presented in Fig. S4. Then, the spacer layer (15 mm × 50 mm) with a square (10 mm × 10 mm) was fabricated by laser engraving the BC film using a 10.6-µm CO_2_ infrared laser under ambient condition and brushed with eco-friendly starch glue for interfacial coupling. Finally, sensing layer was stacked with spacer layer and sandwiched between the encapsulation layers, followed by a pressing process using a compressor (2 MPa, 5 min) to fabricate the pressure sensor.

### Molecular Dynamics Simulation Parameters for Self-Assembly Dynamics of MXene-Cellulose Composites

MD simulations were performed using the LAMMPS [1] software package with a 1-fs time step to study the self-assembly of MXene and cellulose composites during vacuum filtration. The OPLS-AA [2] force field described cellulose molecules, while the UFF [3] potential was applied to MXene nanosheets with surface functional groups (60% hydroxyl, 20% oxygen, 20% fluorine). Water molecules were modeled using the SPC/E [4] model, and interactions were described by LJ and Coulombic forces, with the PPPM method for long-range interactions. A simulation box containing 10 MXene nanosheets, 50 cellulose molecules, and 2000 water molecules was constructed. Each cellulose molecule consisted of 4 glucose monomers, and each MXene nanosheet had a side length of 2 nm, with periodic boundary conditions in xy directions and non-periodic in z direction. After structure optimization, a 500-ps relaxation under the NVT ensemble at 300 K was performed using the Nosé–Hoover thermostat. The vacuum filtration process was simulated with shear deformation in the *xz* direction (1 ns^−1^) and a -*z* directional force of 0.01 (kcal/mol)/Å over 5 ns, maintaining 300 K. Self-assembly dynamics were characterized by analyzing molecular heights, spatial distributions, and alignment.

### Characterization

Atomic force microscope (AFM) imaging was performed with a Bruker Multimode 8 instrument (Germany). The transmission electron microscopy (TEM) imaging was conducted on a JEM-F200 at 80 kV. XRD patterns were recorded on the Bruker D8 Advance Diffractometer equipped with Cu Ka radiation (*λ* = l.5406 Å). Fourier transform infrared (FTIR) spectra were recorded across 400–4000 cm^−1^ (Nicolet iN10-MX). The X-ray photoelectron spectroscopy (Thermo Scientific K-Alpha) with Al Kα radiation was used to examine the bonding interactions. The contact angles (CAs) were obtained via optical CA measuring device (OCA20). Scanning electron microscopy (SEM) images and energy-dispersive spectroscopy (EDS) were collected using a field emission scanning electron microscope (Hitachi SU8010). The optical morphological observation was measured via Olympus LV320 polarizing microscope. The average surface roughness and 3D profilometry measurements were performed with a KEYENCE VK-X150 laser scanning confocal microscope. The conductivity was obtained by employing a four-point probe method with a Keithley 2450 source meter.

### Physical Characterization and Stability Evaluation

#### Measurement of WVTR

The water vapor transmission rate (WVTR) of sensor at 20 and 37 °C was assessed following the ASTM E96-95. Measurements were performed by periodically recording the water weight loss from a bottle sealed with the sensor. WVTR values were calculated using the following equation:

1 $${\mathrm{WVTR}} = \frac{G/t}{A}$$

where *G* represents the weight change, *t* denotes the duration of weight change, and *A* refers to the test area of the sample.

#### Structural Integrity Investigation

Puncture resistance was evaluated on a universal material tester (Zwell/Roell) using a sample holder (diameter: 100 mm) and a steel needle (radius: 450 μm). Penetration was carried out at a speed of (50 ± 5) mm min^−1^ to determine the maximum load.

#### Degradation Profile

Samples for PBS degradation tests were prepared as disks (~ 4 cm in diameter) and incubated at 37 °C in PBS (pH 7.4, Mn = 72,300 g mol^–1^) to evaluate their degradation behavior. For biodegradability tests, the sample (diameter 4 cm, thickness 120 μm) was buried in soil (≈ 30 °C, campus of Beijing Forestry University) and the morphology variations were continuously recorded via digital camera as a function of time.

#### Signal Distortion Assessment

The contact impedance under cyclic bending was measured by a horizontal tension machine in tandem with electrochemical workstation (Autolab) with constant voltage (1 V) for signal acquisition. The humidity and temperature variations were provided by a humidifier and heater in a closed environment, and the wires were protected with waterproof tape.

## Results and Discussion

### Design and Configuration for Respiratory Rate Monitoring Sensor

**Fig. 1:**
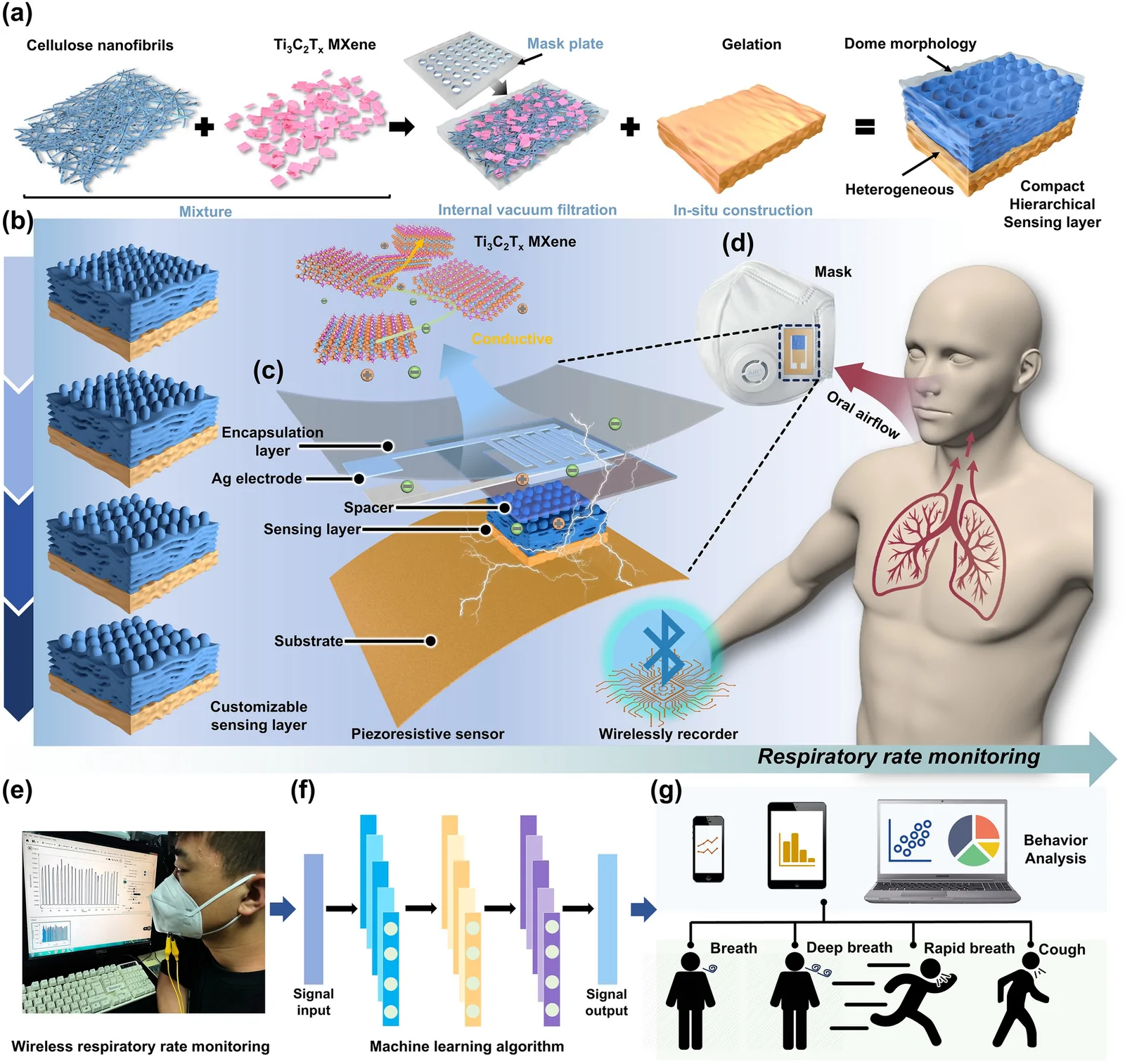
Overall designing strategy. **a** Preparation process of the sensing layer. **b** Schematic diagram of customizable cylindrical interface structure on sensing layer. **c** The composition overview of the pressure sensor and the response mechanism. **d** Schematic diagram of the sensor for respiratory rate monitoring. **e** Photographs of the sensor integrated on the mask to continuous monitor respiratory status. **f** The machine learning algorithm for breathing status classification. **g** Envisioned application for distinguishing various respiratory conditions (including normal breath, fast breath, deep breath, and cough) on mobile terminal devices

To provide ultra-sensitivity, interference-free performance, and structural stability in a piezoresistive sensor paradigm (CPS), a cellulose integrated sensing layer (hereinafter referred to as CISL) is meticulously engineered using a vacuum-assisted self-assembly method for the customizable preparation of cylindrical microstructure. Accordingly, a series of self-assembled, compact, laminated MXene/TEMPO-oxidized cellulose nanofibrils (TOCNF) wet cakes, are primarily fabricated on a porous substrate using different high-resolution laser-engraved mask plate (aperture diameter: 100, 200, 300, and 500 μm) and filtration at 0.1 MPa for 6 h, forming a unique multi-layer in-plane orientation and dome-shaped micromorphology as conductive layer [32, 33]. To further enhance the mechanical integrity of CISL, a gelatin layer can be formed in situ as a robust layer to couple with conductive layer via abundant interfacial bonding and thus contributes to a compact hierarchical laminated architecture for CISL. Notably, the abundant “dynamic bridges” forming internal connections enhance mechanical integrity and facilitate hierarchical structure construction (Figs. 1a, b and S1, S2). It should be noted that, in sharp contrast to conventional polymer-based sensors, sustainable and mechanically robust cellulose nanofibrils and successfully prepared MXene are deliberately integrated as the building block for scalable and customizable architectural design, which also ensures the low-cost and simple manufacturing of CISL, along with excellent biodegradability and biocompatibility (Fig. S3).

To explore the piezoresistive response enabled by reliable ohmic interconnections, Ag interdigital electrodes (10 mm × 57 mm) are patterned onto cellulose paper using a screen-printing technique with surface spraying (Figs. S4 and S5). It is worth emphasizing that the screen-printing process begins with a cellulose-based encapsulation layer (BC film) applied as the substrate. Conductive Ag-based ink is then deposited using a mask-defined stencil, followed by vacuum degassing and curing to ensure both high conductivity and structural stability. Finally, the fully biodegradable electrodes are integrated with the sensing layer, spacer, and encapsulation layer, creating a cohesive and reliable cellulose-based piezoresistive sensor (CPS) for expiratory pressure monitoring (Fig. 1c, d). Notably, the assembled device with sensing layer design achieves collection features that can be applied to continuous respiratory rate monitoring and transmit real-time data wirelessly via bluetooth to an external recorder (Figs. 1e and S6) [34, 35]. By further analyzing the artifact-free output electrical signal with machine learning algorithms, the CPS-based respiratory rate monitoring mask enables real-time monitoring and distinguishing various conditions, including normal breathing, deep breathing, rapid breathing, and coughing, reflecting the body’s real-time health status (Fig. 1f, g).

### Design Strategy and Configuration

**Fig. 2:**
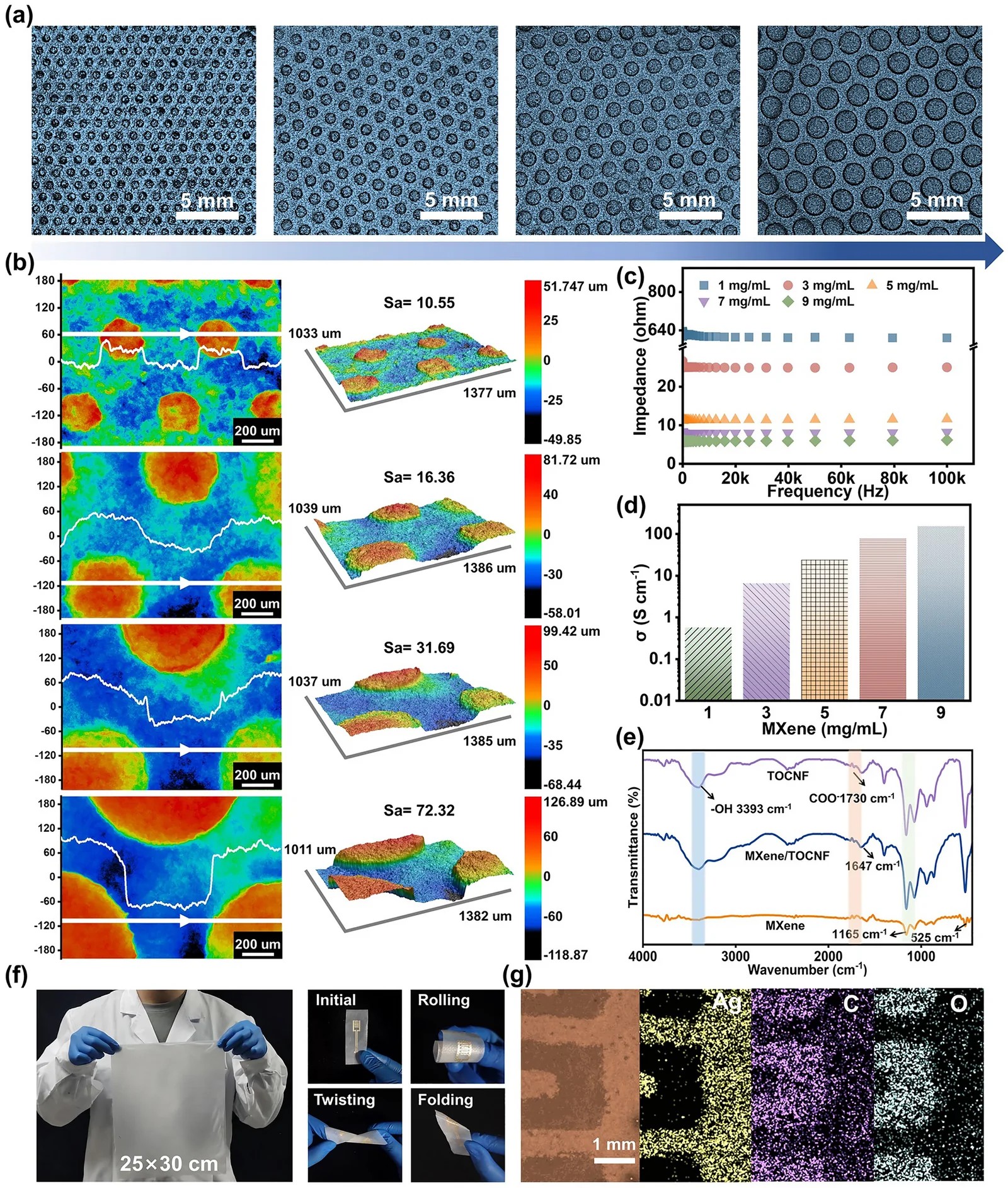
Architecture design and preparation. **a** Photographs of sensing layer with the increased cylindrical dimension. Scale bars, 5 mm. **b** Surface roughness and topographical profiles of sensing layers. **c** Surface impedance measurements of the sensing layer with different MXene concentrations. **d** Electric conductivity (σ) and e the resistance variations over 30 days of sensing layer with different concentrations of MXene (1, 3, 5, 7, and 9 mg mL^−1^). **e** FTIR spectra of the MXene nanosheets, TOCNF, and MXene/TOCNF, respectively. **f** Large-area encapsulation layer (BC film, 25 cm × 30 cm) and flexibility demonstrations of the assembled electrode, such as twisting, folding, and rolling. **g** SEM image and EDS mapping images (Ag, C, and O) of screen‐printed Ag‐based interdigitated electrode on outer encapsulation layer

To optimize piezoresistive performance, it is essential to consider the microstructural design of sensing layers with tunable dome architecture [36–39]. With these aims in mind, a systematic exploration is necessary to understand the underlying design principle. Herein, we employed masks of varying sizes to fabricate sensing layers with tunable cylindrical microstructure dimensions. Figure 2a reveals well-aligned cylindrical domes with remarkable uniformity and consistency, ensuring structural stability and minimizing localized stress points. This configuration promotes reproducible piezoresistive responses, directly correlating the engineered microstructure with enhanced functional attributes. Moreover, the cylindrical dome geometry is selected for its reproducibility through mask-assisted filtration and its ability to provide a stable and sufficiently large contact area during deformation, which helps broaden the response region and enhance pressure sensitivity. In order to investigate the relationship between surface roughness and sensor performance, a series of surface roughness mappings and 3D topographical profiles are constructed and analyzed (Fig. 2b). The results clearly verify that the progressive increase in surface roughness (Sa) from 10.55 to 72.32 µm, corresponding to larger mask sizes, leads to the pronounced surface undulations.

For the parameter optimization, the effect of MXene concentration on the surface impedance behavior is primarily investigated (Fig. 2c). As the MXene concentration increases from 1 to 9 mg mL^−1^, the impedance decreases and stabilizes across all frequencies, which is attributed to the formation of dense and interconnected nanosheet networks that facilitated efficient electron transport. Besides, the CISL sensing layer also exhibits enhanced electric conductivity (σ) with increasing MXene content (Fig. 2d). We clearly observe the significantly improved conductivity and reduced resistance, as visualized by the enhanced brightness of the “BJFU” LED (Fig. S7). Notably, the resistance stability of the sensing layer at 9 mg mL^−1^ MXene concentration exhibits low initial resistance and excellent long-term stability (Fig. S8). Besides, the puncture resistance and mechanical properties of sensing layer are also improved with increasing cellulose concentration (Figs. S9 and S10). The sample (0.16 wt%) achieves a puncture force of approximately 4.0 N, 1.67 times higher than the low concentration sample (0.08 wt%). Also, the elastic modulus and tensile stress reach 24 and 50 MPa, respectively, while toughness exceeds 65 MJ m^−3^, indicating superior energy dissipation for withstanding external force.

The above components offer great opportunity for performance optimization of conductive layer in CISL via abundant dynamic interactions. As illustrated by FTIR spectroscopy (Fig. 2e), the O–H stretching vibration (3393 cm^−1^), the C=O stretching vibration (from 1730 to 1720 cm^−1^), a new low-intensity peak at 1647 cm^−1^ collectively indicate the hydrogen bonding between the two components (Note S1), thus providing strong evidence of TOCNF and MXene for desirable structural integrity. Indeed, the XRD patterns reveal the characteristic (101) and (002) crystalline peaks of cellulose I, confirming the crystalline structure of cellulose (Fig. S11). The conductive layer in CISL exhibits a prominent (002) peak, which shifts from 9.3° to 7.2° with increasing TOCNF content, indicating potential interactions between MXene and TOCNF. Additionally, the encapsulation layer and screen-printed Ag electrode maintained operational stability under extreme deformation [15, 40–42]. The prepared large-area cellulose film (25 cm × 30 cm) can be seamlessly integrated with irregular surfaces and deformed, i.e., rolling, twisting, and folding (Figs. 2f and S12). To elucidate the uniformity and distribution of the electrode, SEM and energy-dispersive spectroscopy (EDS) analysis images are performed, validating that the Ag-based interdigitated electrode is uniformly distributed on the bacterial cellulose layer without any aggregation, and the Ag localized specifically within the interdigitated regions (Fig. 2g). The above results indicate the high-quality fabrication process and the compatibility of the electrode with the BC encapsulation layer, reinforcing its potential for flexible, durable, and high-performance piezoresistive sensing applications.

### Structure Arrangement and Assembly of Sensor

**Fig. 3:**
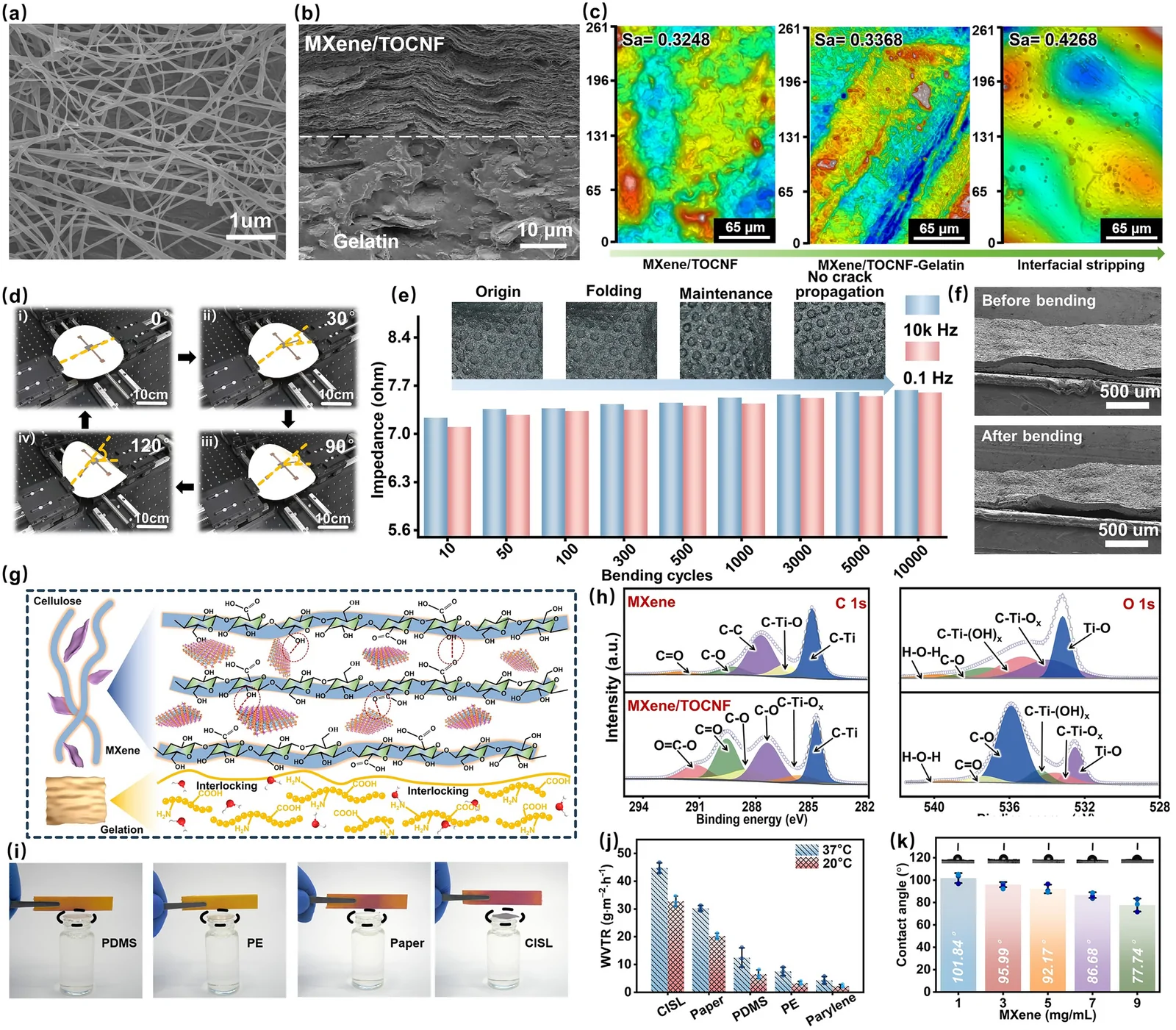
Dynamic interactions and collection merits. SEM image of **a** highly interwoven cellulose network structure cellulose and **b** the compact laminated heterogeneous sensing layer. **c** Surface roughness of MXene/TOCNF, MXene/TOCNF-Gelatin, and the sensing layer after interfacial stripping. **d** Photographs of continuous bending tests with different angles via horizontal tensile machine. **e** Impedance variation of sensing layer even after 10,000 folding fatigue cycles (radius 2 cm, angle 180°), the inset images showing the interface without crack propagation. **f** Cross-section SEM images after 10,000 bending cycles without structural destruction. **g** Schematic illustration of dynamic interactions between MXene and cellulose-gelation for architecture construction. **h** The corresponding C 1*s* and O 1*s* XPS spectra of MXene nanosheets and MXene/TOCNF. **i** Permeability verification via the hydrochloric acid permeates the sensing layer and discolored the pH indicator. **j** Water vapor transmission rate (WVTR) of the sensing layer in comparison with paper, PDMS, PE, parylene at 20 and 37 °C. **k** Water contact angle of sensing layer with different concentrations of MXene (1, 3, 5, 7, and 9 mg mL^−1^, respectively). Error bars represent the s.d. of the mean from 3 samples

In light of intuitively elaborating the architecture design, we present a comprehensive characterization of the MXene/TOCNF-gelation sensing layer (CISL), demonstrating its exceptional structural, mechanical, and functional properties. Figure 3a shows that the TOCNF can achieve a uniform and interconnected network via dynamic bonds, which can collectively serve as an ideal matrix for reinforcing the sensing layer. The cross-sectional SEM image (Figs. 3b and S13) distinctly reveals a compact laminated heterogeneous sensing layer, highlighting an intimate interface formed between gelatin and MXene/TOCNF layers. The 3D surface topography images in Figs. 3c and S14 clearly illustrate significant morphological differences among the MXene/TOCNF, MXene/TOCNF-Gelatin, and interfacial stripping samples. The first two images show a rough, fibrous, and interwoven surface, indicating the presence of a complex interfacial microstructure. The addition of gelatin appears to enhance interfacial coupling through molecular-level interactions, as evidenced by the increased surface roughness (Sa = 0.3368). In contrast, the interfacial stripping image demonstrates a high roughness (Sa = 0.4268), suggesting that the interface of sensing layer after stripping has significantly destruction because of the effective interfacial coupling.

As depicted in Fig. 3d, continuous bending tests (10,000 cycles) are conducted to evaluate the mechanical stability of robust heterogeneous CISL. Remarkably, the CISL exhibits minimal impedance variation at 10 kHz and 0.1 Hz even after 10,000 folding fatigue cycles, with a bending radius of approximately 2 cm and an angle of around 180°, highlighting the ability to maintain its electrical properties under cycle deformation conditions (Fig. 3e). The inset images further demonstrate that the interface remains compact and free from delamination or physical damage, which mainly ascribed to the abundant hydrogen bonds and layer-by-layer energy dissipation in laminated heterogeneous sensing layer. Figure 3f further substantiates these findings, with cross-sectional SEM images before (top) and after (bottom) the 10,000 bending cycles. The images reveal that the sensing layer remains intact and free from visible damage or delamination, even after extensive cycling. Based on the above investigation, it is evident that the CISL exhibits exceptional durability and maintains both mechanical integrity and electrical functionality [43, 44]. To elucidate the underlying mechanisms, Fig. 3g illustrates the dynamic interactions between MXene nanosheets, cellulose nanofibrils, and gelatin. On the premise of MXene nanosheets guarantee the excellent electrical conductivity, the dynamic bonding enables the cellulose to serve as a structural scaffold, enhancing the mechanical strength and flexibility. The FTIR spectra (Fig. S15) provide clear evidence for strong interfacial coupling between MXene/TOCNF and gelatin. Compared to MXene/TOCNF, the MXene/TOCNF-Gelatin composite exhibits characteristic peaks at 1540 cm^−1^ (amide II, N–H bending/C–N stretching) and 1235 cm^−1^ (amide III), confirming successful gelatin incorporation. Additionally, the attenuation and shift of the COO^−^ stretching vibration at 1730 cm^−1^ indicate robust hydrogen bonding and electrostatic interactions at the MXene/TOCNF-Gelatin interface. To this end, the chemical interaction is further investigated by analyzing the C 1*s* and O 1*s* core-level spectra (Fig. 3h). In the high-resolution C 1s spectrum of MXene/TOCNF, a noticeable positive shift in the C–O peak to 286.4 eV is observed, further suggesting the presence of multiple hydrogen bonds between MXene nanosheets and TOCNF. For the O 1*s* spectrum, the decomposition of the MXene spectrum reveals five peaks at 539.6, 537.4, 535.9, 534.4, and 533.6 eV, corresponding to Ti–O, Ti–C–O_X_, Ti–C–(OH)_X_, C–O, and H–O–H bonds. In the MXene/TOCNF composite, the O 1s spectrum is decomposed into six peaks at the same binding energies, with the addition of a new peak at 532.5 eV attributed to C=O bonds. This new peak indicates the involvement of additional oxygenated functional groups from TOCNF in the interaction with MXene. The slight up-shift in the O 1s binding energy in the MXene/TOCNF composite compared to pure MXene further supports the formation of hydrogen bonds between the MXene nanosheets and TOCNF.

Additionally, we find that the CISL demonstrates significant permeability, as evidenced by the successful penetration of hydrochloric acid from the sample vial, which resulted in a visible discoloration of the pH indicator (Fig. 3i). The water vapor transmission rate (WVTR) of CISL shows a substantial increase at various conditions (20 °C) and sweaty (37 °C), from approximately 615 to 4424 g m^−2^ d^−1^, indicating superior moisture permeability compares with that of paper, PDMS, PE, and parylene (Fig. 3j). This improved performance confirms that the CISL can facilitate efficient moisture transfer for preventing moisture accumulation-enabled signal fluctuations. Moreover, owing to its bio-based composition, the CISL demonstrates controlled degradation in 4 wt% H_2_O_2_ solution at room temperature (Fig. S16). The material maintains structural integrity at 0.5 h but gradually disintegrates by 12 and 24 h, achieving complete breakdown within 72 h, indicating its environmentally friendly disposal potential. Within 30 days, significant decomposition of CISL is evident, and by 150 days, near-complete degradation is observed (Fig. S17). In contrast, PDMS remains intact throughout the 150-day period, indicating minimal biodegradability. These results underscore the eco-friendly nature of CISL, offering a sustainable alternative to conventional synthetic materials for practical, environmentally responsible applications [45]. Besides, water contact angle (WCA) tests of CISL are performed at various MXene concentrations (Figs. 3k and S18). It can be observed that as the MXene concentration increased, the WCA progressively decreased from 101.84° to 77.74°, illustrating the increasing affinity of the surface for water, driven by the enhanced exposure of polar functional groups, such as hydroxyl and carboxyl groups, on the MXene surface.

### Microscopic Mechanism of Shear Force-Steered Structure Construction

**Fig. 4:**
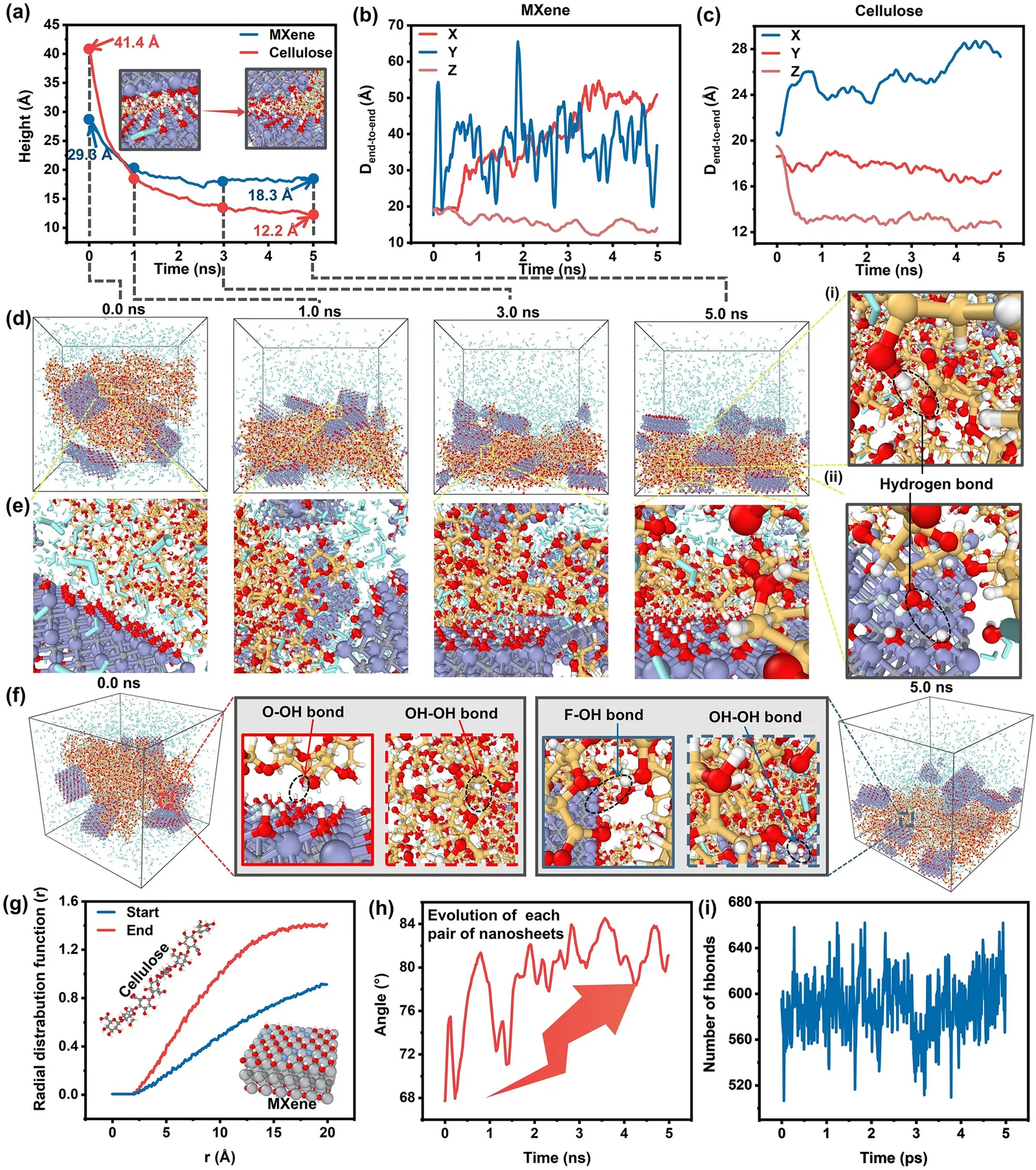
Theoretical analysis of architecture fabrication. Evolution process of MXene nanosheets and cellulose nanofibrils within aqueous solution under shear force fields: **a** average height of MXene and cellulose molecules over time. **b**, **c** End-to-end distance (*D*_end-to-end_) of MXene and cellulose molecules along the *X*-, *Y*-, and *Z*-axes over time, respectively. Among them, *D*_end-to-end_ refers to the distance between the C atoms at the two edges of the polymer molecules. **d** Simulation configurations under shear force fields at 0, 1, 3, and 5 ns are presented. **e** Locally MDr dynamics snapshots. **f** Molecular cage configurations at 0 and 5 ns in different directions and their locally magnified snapshots. **g** Radial distribution function at the start and end systems. **h** Evolution process of the sharp angles between MXene and cellulose within aqueous solution under shear force fields. **i** The number of hydrogen bonds formed between MXene and cellulose within 5 ns

Vacuum filtration technique has been widely reported as a low-cost and efficient material preparation strategy in the literature, while still remains a largely unexplored for the underlying mechanism. To further probe the self-assembly process of MXene/cellulose conductive layer for CISL during vacuum filtration, MD simulations are performed using the LAMMPS software package [46–49]. A simulation box containing 10 MXene nanosheets, 50 cellulose molecules, and 2000 water molecules is constructed to analyze the structural organization and interactions within the composite system, providing insights into the dynamics and driving forces of the assembly process. As exemplified in Fig. 4a, the average heights of MXene and cellulose molecules decrease systematically over time during the self-assembly process, from initial positions of 41.4 and 29.3 Å to 18.3 and 12.2 Å, respectively [50]. This reduction reflects effective sedimentation driven by shear and filtration forces, promoting molecular alignment and the formation of a compact nanocomposite structure. Contrary to the common perception that vacuum filtration induces only vertical sedimentation, the simulation reveals shear forces, also a main factor to generate rotational stacking, resulting in unique molecular arrangements. The insets further highlight the structural reorganization over time, confirming the progression of the self-assembly process. The alignment behavior of MXene and cellulose is analyzed through their end-to-end distances (*D*_end-to-end_) (Fig. 4b, c), showing a strong preference for alignment along the *x*-axis, where the distances exhibit a distinct pattern specific to this direction. In contrast, the *y*-axis and *z*-axis exhibit consistent distances, further emphasizing the directional orientation that contributes to the formation of a well-ordered structure [51, 52].

In the initial configuration (0 ns), MXene and cellulose molecules are randomly dispersed throughout the simulation box (Figs. 4d and S19, Video S1). Under shear forces, the molecules begin to aggregate and settle over time. By 1 ns, initial sedimentation is observed, with both MXene and cellulose moving toward the bottom. At 3 ns, significant alignment and compaction occur, and by 5 ns, a dense, well-ordered layer formed at the bottom of the simulation box. Figure 4e provides magnified snapshots of these intervals, revealing the evolving molecular interactions. At 5 ns, the insets show the formation of hydrogen bonds between MXene and cellulose molecules, emphasizing their strong interfacial interactions, which play a key role in stabilizing the composite structure. A more detailed mechanistic evolution is shown in Fig. 4f. Initially (0 ns), MXene and cellulose molecules are in an unstructured state due to weak intermolecular interactions and a lack of directional alignment [47, 53]. The insets reveal O–OH and OH–OH hydrogen bonds, representing initial bonding between MXene functional groups and cellulose. In sharp contrast, by 5 ns, the molecules transition to a dense, well-organized state at the bottom of the simulation box, driven by the combined effects of shear forces and gravitational settling. The insets at 5 ns highlight additional F–OH bonds between fluorinated MXene and cellulose, alongside persistent OH–OH bonds. These enhanced interfacial interactions stabilize the composite and contribute to its mechanical integrity.

In addition, the interactions within MXene/cellulose nanocomposites are analyzed using radial distribution functions (RDFs) (Fig. 4g). The RDF curves depict the changes in the distance between MXene and cellulose molecules during the simulation. Initially (blue curve), the RDF values reflect weak interactions and a notable separation. By the end of the simulation (red curve), the RDF values at short distances reflect interactions with a close spatial arrangement. The inset highlights the structural arrangement of MXene and cellulose molecules during the simulation. Shear forces drive the gradual alignment of MXene and cellulose over the 5-ns simulation (Fig. 4h) [40]. In the early stages, molecular angles fluctuate, reflecting a disordered state. By 2 ns, partial alignment begins, though inconsistencies remain. After 3 ns, the angles stabilize near 80°, indicating improved alignment. Minor fluctuations persist, likely due to localized rotational motion or aggregation, underscoring the dynamic nature of the self-assembly process. Finally, the number of hydrogen bonds between MXene and cellulose molecules, shown in Fig. 4i, remains relatively stable throughout the 5-ns simulation, fluctuating between 520 and 640. Contrary to expectations that vacuum filtration would increase hydrogen bonding and crosslinking density, the simulation reveals no significant change. This suggests that hydrogen bonds contribute to interfacial stability but are not the primary drivers of molecular reorganization. Instead, van der Waals forces likely dominate alignment and densification during self-assembly. These findings highlight the need to optimize hydrogen bonding and crosslinking density to enhance structural properties under similar conditions. The stability of hydrogen bonds underscores their role in maintaining structural integrity while enabling dynamic rearrangements under applied forces.

### Comprehensive Evaluation for Pressure-Based Respiratory Rate Monitoring

**Fig. 5:**
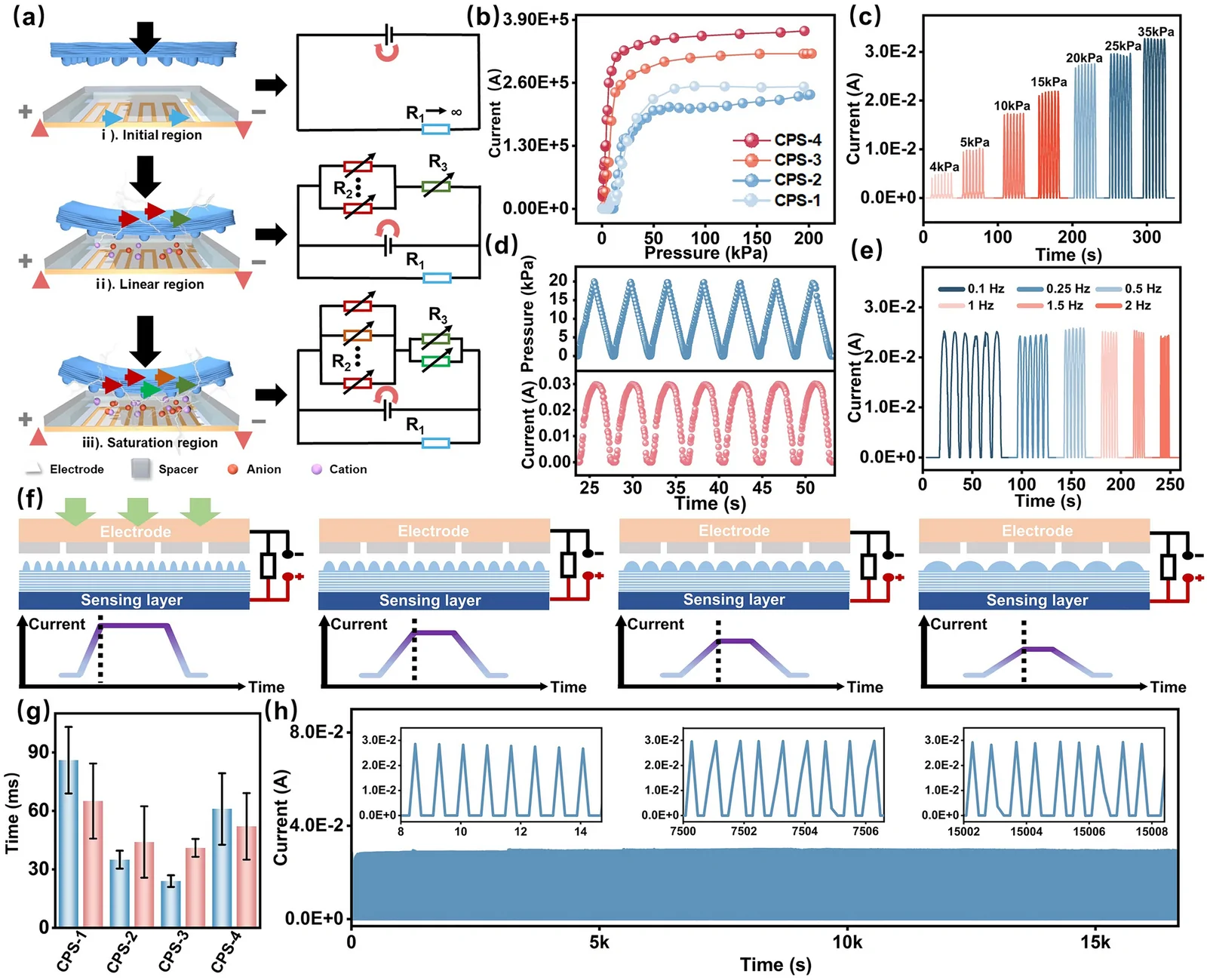
Piezoresistive response principle. **a** Schematic illustration of the sensing mechanism and the corresponding equivalent circuit. **b** Sensitivity curve of the CPS with different cylindrical interface dimensions (0–200 kPa). **c** The accurate piezoresistive response in the range of 4, 5, 10, 15, 20, 25, and 35 kPa, respectively. **d** The negligible hysteresis between pressure and output signals. **e** The stable sensing under different response frequency (0.1, 0.25, 0.5, 1, 1.5, and 2 Hz, respectively). **f** The I-T curve for different cylindrical interface dimensions. **g** Responding/recovery time of the CPS. **h** Cyclic stability of CPS pressure sensor over 25,000 cycles (~ 20 kPa). Inset: magnified resistance variations over a 10-cycle period

As scheme depicted in Fig. 5a, the sensitivity curve of CPS based on CISL can be divided into initial region, linear region, and saturation region with the aid of simplified equivalent circuit model. Specifically, in the initial region, the dome-shaped sensing structure maintains minimal contact with the electrodes, resulting in a high total resistance. With applied load, the contact area increases, transitioning into the linear region. In the saturation region, full compression maximizes the contact area, making the primary contributor to resistance changes. To quantitatively decipher the sensitivity and current response of the CPS under varying cylindrical interface dimensions (named as CPS-1, CPS-2, CPS-3, and CPS-4), Fig. 5b evaluates the size-dependent behavior. The sensitivity curve at 200 kPa shows a linear region with proportional pressure response and a saturation region where sensitivity plateaus due to compression-induced limitations. CPS-4 enhances sensitivity by providing an extensive contact area and strain distribution, while CPS-1 maintains a wide linear range, ensuring stability across a diverse pressure spectrum. Besides, Fig. 5c exhibits that the CPS can achieve obvious and stable signal variation under external pressures (from 4 to 35 kPa), further suggesting that the microstructure design contributes to high sensitivity for helping to identifying the subtle pressure variation of the exhaled gas. Furthermore, the high degree of synchronization between current and pressure curves proves the low hysteresis of signal outputs against the fast and irregular dynamic breathing conditions (Fig. 5d). Similarly, such a high sensitivity and stability can be maintained even under different pressure response frequency from 0.1 to 2 Hz (Fig. 5e). Complementing this, Fig. 5f further reveals that CPS-1 produces prominent current peaks and substantial responses due to localized pressure concentration and significant strain. In contrast, CPS-4 exhibits low current peaks and focused responses due to uniform pressure distribution and minimized strain. These results quantitatively prove that the architecture design effectively solves the trade-off between sensitivity and response stability, emphasizing the importance of optimizing interface dimensions for specific application requirements.

More interestingly, the dynamic response of CPS is investigated and found that the response and recovery times decrease from 86 and 65 ms to 24 and 41 ms, respectively, in the case of elevating detection interface dimensions from the CPS-1 to the CPS-4 (Figs. 5g and S20). According to the results, smooth current transitions and rapid stabilization are achieved with expanded interfaces, indicating that the improved contact area and strain redistribution are consistent with the reduced response and recovery times under the same conditions. Besides, the enhanced electrical stability and rapid performance reflect the ability to achieve efficient contact and separation. As further validation, the CPS demonstrates remarkable stability and adaptability across varying cycles. With an cycles of 50, it achieves a steady-state current of 9.98 × 10^–6^ A, equivalent to 1.02 times the value observed at an cycles of 1. This outcome is attributed to enhanced strain transfer and contact efficiency (Fig. S21). Dynamic current profiles under cyclic loading show consistent peaks, with large cycles delivering high amplitudes owing to improved strain distribution and deformation. To be more intuitively, the cyclic stability of CPS is evaluated over 25,000 loading/unloading cycles under 20 kPa (Fig. 5h), demonstrating highly consistent current responses with no observable drift or amplitude degradation. The magnified insets of periods confirm stable signal intensity and waveform fidelity at the start, midpoint, and end of the test, underscoring the extraordinary electrical reliability and structural integrity. These results collectively verify the excellent stability for withstanding complex deformation in real-time respiratory rate monitoring. In the context of significantly enhancing the comprehensive performance, the radar plot demonstrates the superior capabilities compared to previously reported pressure sensors. The CPS combines high sensitivity, broad range, fast response, and stability for precise pressure monitoring (Fig. S22, Table S1, and Note S2) [7, 13, 22, 27, 35, 54]. Its customizability, biodegradability, and durability under harsh conditions make it ideal for sustainable and flexible applications.

### Validation of Accurate Signal without Distortion

**Fig. 6:**
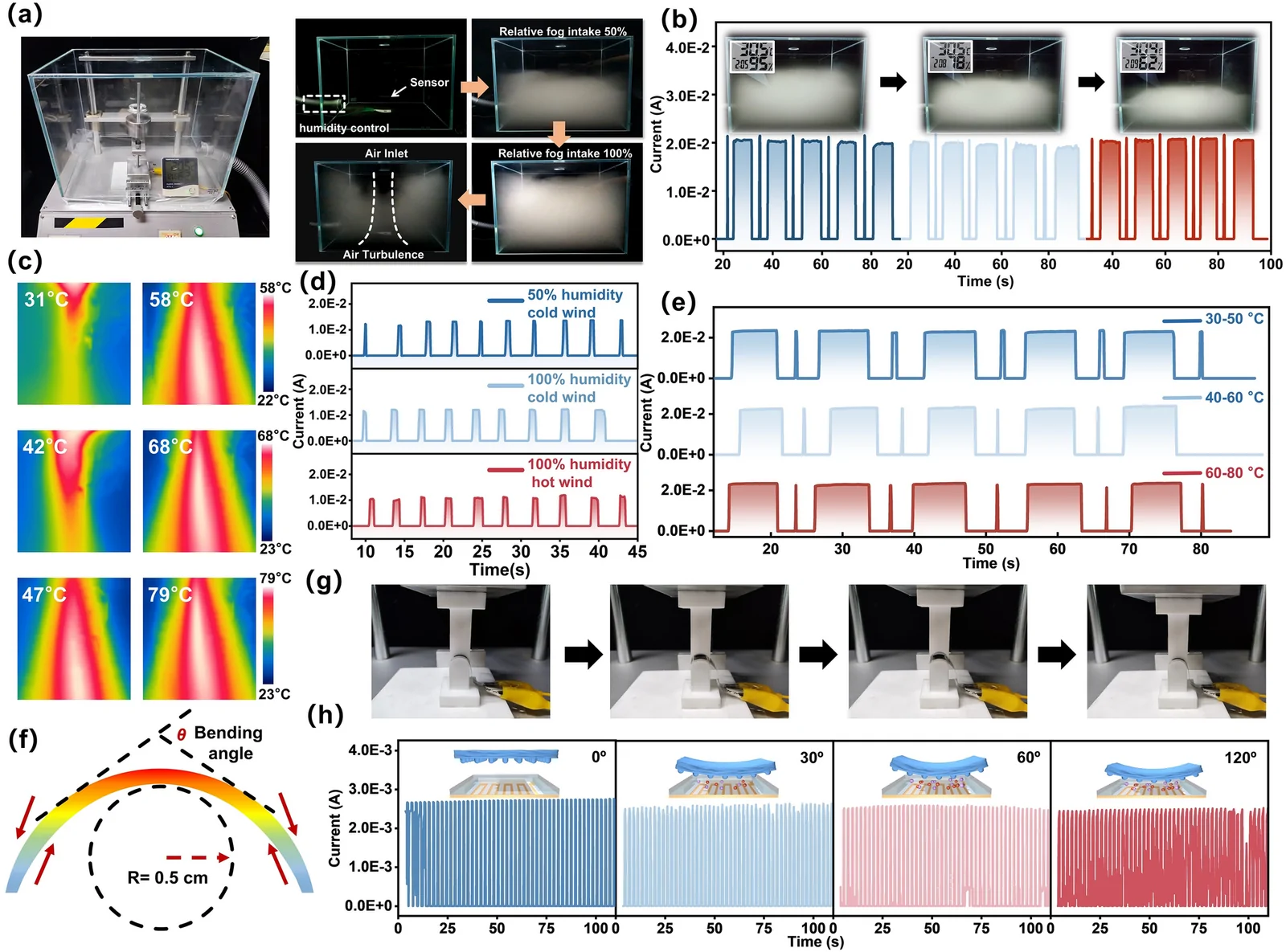
Stability performance of CPS under multiple signal inputs. **a** Photographs of humidity control system with a glass fog chamber and the humidity change process. **b** I-T curves of CPS without obvious fluctuation at different humidity (95%, 78%, and 62%). **c** IR camera images of airflow disturbance at different temperatures (31, 42, 47, 58, 68, and 79 °C). **d** Steady I-T curves for various multi-signal input conditions (50% humidity and cold wind, 100% humidity and cold wind, 100% humidity and hot wind). **e** I-T curves of CPS corresponding to different temperature ranges (30–50, 40–60, and 60–80 °C). **f** Schematic diagram of the stress concentration in bending state (radius = 0.5 cm, angle: 0–120°). **g** Photographs of CPS under various bending. **h** Signal stability under varying bending angles (0°, 30°, 60°, and 120°)

To ensure applicability of respiratory rate monitoring sensor across various environmental conditions, including high humidity, extreme temperature, and mechanical deformation, it must possess excellent moisture resistance, thermal stability, and structural robustness. We thus assemble the equipment to evaluate the cumulative moisture on the stable signal performance of assembled CPS in high-humidity environments (Fig. 6a**, **Video S2). To simulate these conditions, a glass fog chamber is constructed, which allows for controlled moisture intake and airflow. As indicated, the chamber has 40-mm holes on the left and top, facilitating the intake of fog and the circulation of air. The CPS is positioned directly below the air blowing hole to expose it to the humidified air, thereby mimicking the varying humidity levels. Notably, the CPS maintains stable performance (~ 2.07 × 10^–2^ A) across varying humidity levels of 95%, 78%, and 62%, demonstrating its ability to function consistently under different humidity gradients (Fig. 6b). Subsequently, we test the sensing response of CPS upon temperature variations (31, 42, 47, 58, 68, and 79 °C). The thermal airflow disturbance in infrared thermal images clearly reflects the hot air and is visualized by color gradients showing the air temperature changes (Figs. 6c and S23). Specifically, the temperature variations are highly consistent with the increasing airflow temperature, as evidenced by the gradual expansion of the red and yellow regions from 31 to 79 °C, that confirming the controllability of temperature variations. In order to simulate the real usage environment, hot wind is used to trigger sensor operation (Fig. 6d). The system enables coupling control of both humidity and temperature factors (50% humidity and cold wind, 100% humidity and cold wind, 100% humidity and hot wind) and the corresponding I-T curves (~ 1.2 × 10^–2^ A, 10 cycles) without significant fluctuations. This stability stems from both the laminated architecture design and contact-separate sensing mechanism. Compared with the gel materials, which are prone to charge accumulation due to temperature and humidity changes, the contact-separate mechanism of sensor and highly dense laminated layer of sensing layer may effectively diminish the ionization conduction and leads to the thermal carrier transition in a layer-by-layer manner.

Based on these findings, continuous pressure response testing is further conducted under various temperature gradients (30–50, 40–60, and 60–80 °C) in Figs. 6e and S24, Video S3. The corresponding I-T curves illustrate the CPS delivers stable performance (~ 2.0 × 10^–2^ A) and continuous response (90 s) over a specified period under varying environmental conditions, reflecting the robustness and adaptability to satisfactorily guarantee the daily applications. Furthermore, the wearable sensor demonstrates excellent environmental adaptability, maintaining a stable resistance (approximately 43–46 Ω) across a wide range over 14 days, confirming reliable signal transduction even in fluctuating environments. Additionally, for mimicking large and continuous deformation, the bending tests are examined with a constant bending radius (*R* = 0.5 cm), while the bending angle is varied from 0 to 120° (Figs. 6f, g and S25). Promisingly, the output current curves (~ 2.8 × 10^–3^ A) exhibit some fluctuations across different bending angles (0°, 30°, 60°, and 120°), but show relatively stable and consistent responses at each fixed angle without significant signal degradation, even under prolonged deformation for 100 s (Fig. 6h, Videos S4 and S5). This impressive performance further substantiates the exceptional stability of the device, which is attributed to the compact laminated structure of the sensing layer and the contact-separate sensing mechanism that prevents the intrinsic deformation-enabled baseline drift of signals. Also, the laminated design effectively contributes to dissipate local stress and prevent undesired responses from external non-vertical stress contacts, maintaining conductive networks that are insensitive to the structural failure in large and repeated deformations [55, 56].

### Applicability Assessment in Multi-signal Input Scenario

**Fig. 7:**
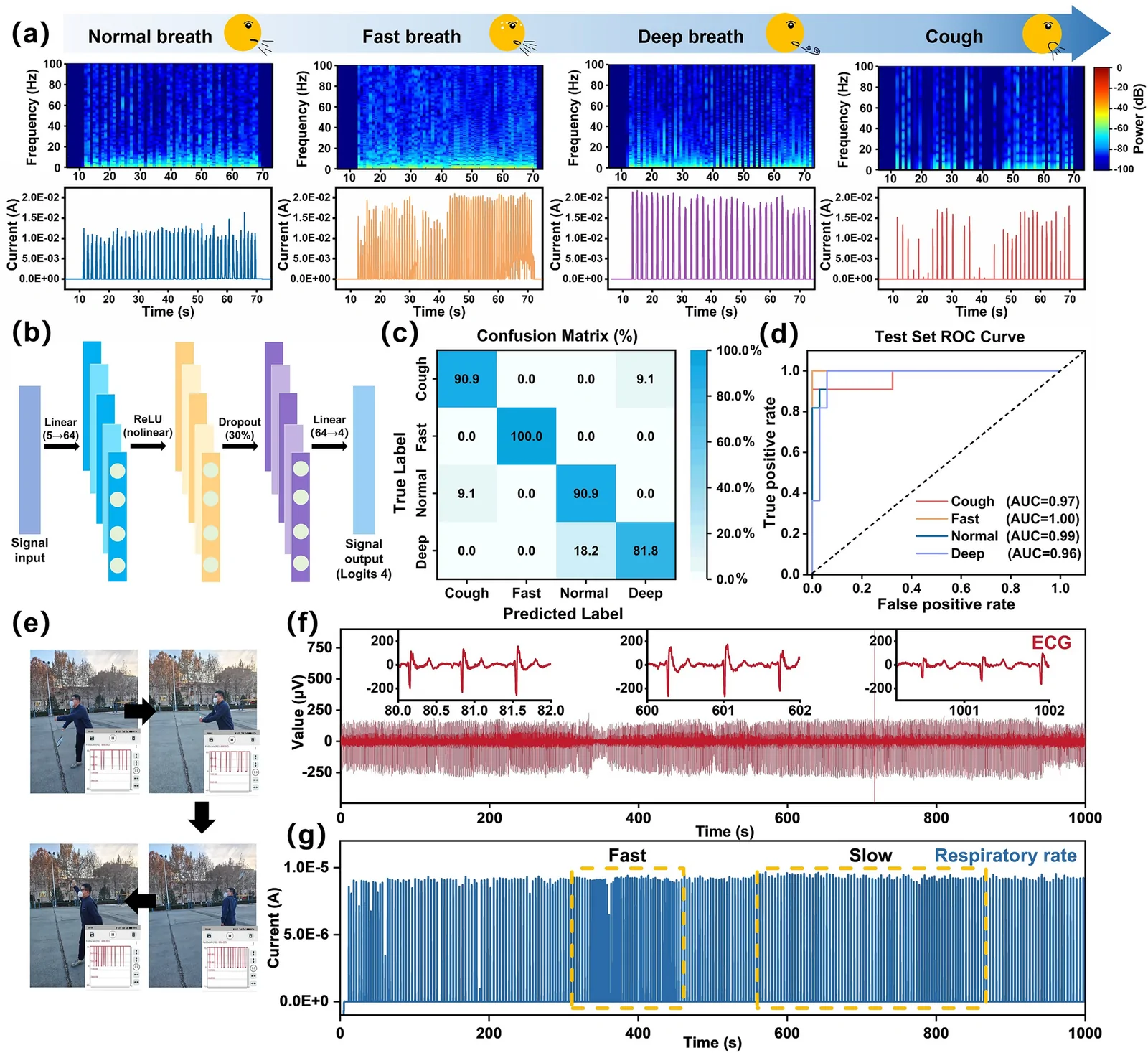
Validation of respiratory rate monitoring. **a** Stable and accurate breathing signals recording via CPS and the corresponding time–frequency domain analysis in terms of normal breath, fast breath, deep breath, and cough conditions. **b** Machine learning algorithm model used for classifying respiratory signal data. **c** Confusion matrix for respiratory pattern classification. **d** ROC curves for the respiratory pattern classification on the test set. **e** Photographs of long-term respiratory rate monitoring over a 20-min duration. **f** Wireless ECG signal recording and **g** the corresponding distinguishable respiratory rate monitoring with obvious degree differentiation

The respiration rate (RRT) can provide critical physiological information, offering insights into cardiac, neurological, and pulmonary conditions, as well as certain other diseases. We thus demonstrate that the integration of customizable CPS on the face mask in combination with the wireless module enables real-time data transmission to monitor dynamic breathing signals. The RRT tests are conducted according to the difference of respiratory intensity in different age groups using a signal acquisition system, integrated with an automatic electrochemical service station and a monitoring display to display real-time feedback (Fig. S26, Video S6). Indeed, the CPS-based face mask can accurately capture the real-time respiration signals without distortion across various breathing conditions, including normal, fast, deep, and cough patterns. The real-time breathing response curves of the four volunteers (Tester I, male, 30-year-old; Tester II, female, 24-year-old; Tester III, male, 30-year-old; Tester IV, female, 27-year-old), analyzed through time–frequency spectra obtained by fast Fourier transform, enable precise analysis of the frequency and amplitude variations in breathing waveforms under different respiratory conditions (Figs. 7a and S27). This analysis clearly reveals the intensity distribution in both the frequency and time domains, highlighting the magnitude parameters and distinguishing the frequency characteristics of motion artifact noise under varying breathing conditions. Typically, for Tester I, the average breath rate and signal intensity during normal, fast, deep, and cough conditions are 44 breaths/min and 0.012 A, 73 breaths min^−1^ and 0.015 A, 33 breaths min^−1^ and 0.02 A, and 34 breaths min^−1^ and 0.015 A, respectively. In comparison, Tester III shows 27 breaths min^−1^ and 0.015 A, 40 breaths min^−1^ and 0.01 A, 23 breaths min^−1^ and 0.01 A, and 20 breaths min^−1^ and 0.013 A across the same conditions. Besides, for Testers II and IV, unique individual patterns emerge, particularly during coughing, with breath rates of 18 breaths/min and 0.009 A and 22 breaths min^−1^ and 0.01 A, respectively. These personalized variations in breathing signals underscore the sensor’s capability to capture subtle individual differences in respiratory behavior. Moreover, abnormal breathing conditions are easily distinguishable from normal breathing, as indicated by the increase in both respiratory frequency and intensity. Collectively, these results corroborate the high sensitivity and reliability of the sustainable CPS, making it a promising tool for long-term and personal RRT monitoring.

To enable accurate recognition of four distinct respiratory states, we designed a deep learning-based classification model to train and classify various breathing conditions. As shown in Fig. 7b, the respiratory signals collected under each condition are segmented and preprocessed using peak detection and periodic slicing and subsequently used to construct a balanced dataset. The data are randomly divided into training set and test set at a 1:1 ratio to evaluate model performance. The confusion matrix in Fig. 7c demonstrated that the model achieved high classification accuracy across all categories, with 100.0% accuracy for fast breathing and 90.9% for both cough and normal breathing. Minor confusion is observed between normal and deep breathing, yet the overall average prediction accuracy reached approximately 90%. To further assess the robustness of the model, ROC analysis is performed. As depicted in Fig. 7d, all classes achieved excellent AUC values, with 0.97 for cough, 1.00 for fast breath, 0.99 for normal breath, and 0.96 for deep breath. It indicated strong discrimination ability and generalization performance. The high accuracy and stability of classification confirmed the feasibility of combining high-resolution sensing with intelligent algorithms for real-time respiratory pattern recognition. To be more intuitively, Fig. 7e demonstrates the real-time RRT monitoring via CPS during badminton game process, simulating real-world scenarios where wearable sensors are obviously subjected to continuous mechanical deformations and multi-signal inputs (temperature, humidity, and bending). The continuous electrocardiogram (ECG) signals over 1000 s in Fig. 7f reflect different physiological states during exercise. The magnified insets at 80–82, 600–602, and 1000–1002 s display clear P-QRS-T waveforms, which correspond closely to changes in respiratory rate under varying physical conditions. This characteristic is further confirmed by long-term respiratory rate monitoring, which reveals a pronounced primary variation between fast (300–450 s) and slow (550–850 s) respiratory rates during exercise scenario (Fig. 7g). Impressively, the closely spaced peaks during fast breathing and widely spaced peaks during slow breathing effectively exhibit the sensor’s high sensitivity and responsiveness to varying respiratory rates. Furthermore, the consistent signal stability and amplitude further highlight the sensor’s robustness and reliability, satisfying with the accurate and real-time RRT monitoring without obvious signal fluctuation.

## Conclusions

In summary, the advancement of sustainable flexible electronics and the urgency of global respiratory disease control accentuate the demand for innovative research endeavors in green material-based respiratory monitoring sensor with desirable signal accuracy and stability. This work pioneers a sensitive and robust respiratory rate monitoring device via scalable cylindrical microstructure design and natural material integration, enabling accurate and stable real-time tracking of breathing states. The system demonstrates high sensitivity and reliability without signal distortion owing to the contact-separate sensing mechanism and robust laminated sensing layer design, achieving clear differentiation of abnormal breathing states based on changes in breath rate and intensity. Notably, the assembled CPS provides remarkable following advances:

1. Compared to existing respiratory rate monitoring sensors, relying on MD and comprehensive characterizations, we verify that the compact laminated design and efficient interfacial bridging (hydrogen bonds) ensure the unique structural stability upon various dynamic scenarios.
2. Capitalizing on the fully cellulose-based materials integration and straightforward patterning achieved through a mask-defined stencil template-assisted vacuum filtration method for architecture design, the sensor possesses desirable customizability and sustainability. It can be degraded of simply by oxidative or soil environments, preventing the accumulation of electronic waste.
3. MD simulations uncover the shear force-driven self-assembly mechanism of MXene/TOCNF nanocomposites during vacuum filtration, addressing a critical gap in understanding dynamic material transitions and molecular interactions. Vacuum filtration-mediated shear forces coupling with vertical sedimentation are identified as key drivers of directional alignment, compaction, and rotational stacking.
4. The substantial progress of respiratory rate measurement via CPS integrated face mask is achieved for accurate distinguishing subtlest changes of breathing among different daily activities (normal, fast, deep breath, and cough).

It can be anticipated that the proposed customizable architecture, simple components configuration, low-cost and scalable preparation method will open up a facile yet economic methodology for high-fidelity health surveillance and paves the way for forecasting the respiratory disorder-derived diseases of vulnerable demographics.

## Supplementary Information

Below is the link to the electronic supplementary material.

Supplementary file1 (MP4 5669 KB)

Supplementary file2 (MP4 1226 KB)

Supplementary file3 (MP4 1594 KB)

Supplementary file4 (MP4 783 KB)

Supplementary file5 (MP4 339 KB)

Supplementary file6 (MP4 755 KB)

Supplementary file7 (DOCX 47204 KB)
