# Supplementary material for: Robust and Biodegradable Heterogeneous Electronics with Customizable Cylindrical Architecture for Interference-Free Respiratory Rate Monitoring
Source: Nanomicro Lett. 2025 Aug 19;18:34. doi: 10.1007/s40820-025-01879-x (PMC12364787; doi:10.1007/s40820-025-01879-x)
Supplement: Supplementary file 7 — Supplementary file7 (DOCX 47204 KB) [file 40820_2025_1879_MOESM7_ESM.docx]

Supporting Information for

**Robust and Biodegradable Heterogeneous Electronics with Customizable Cylindrical Architecture for Interference Free Respiratory Rate Monitoring**

Jing Zhang^1, 4#^, Wenqi Wang^2#^, Sanwei Hao^1*^, Hongnan Zhu^2^, Chao Wang^1^, Zhouyang Hu^2^, Yaru Yu^1^, Fangqing Wang^1^, Peng Fu^4^, Changyou Shao^3*^, Jun Yang^2*^, and Hailin Cong^1*^

^1^ School of Materials Science and Engineering, Shandong University of Technology, Zibo 255000, P. R. China

^2^ Beijing Key Laboratory of Lignocellulosic Chemistry, College of Materials Science and Technology, Beijing Forestry University, Beijing 100083, P. R. China

^3^ Liaoning Key Laboratory of Lignocellulose Chemistry and Biomaterials, College of Light Industry and Chemical Engineering, Dalian Polytechnic University, Dalian 116034, P. R. China

^4^ School of Agricultural Engineering and Food Science, Shandong University of Technology, Zibo 255000, P. R. China

^#^Jing Zhang and Wenqi Wang contributed equally to this work.

*Corresponding authors. E-mail: [haosanweixs@163.com](mailto:haosanweixs@163.com) (Sanwei Hao); [shaocy@dlpu.edu.cn](mailto:shaocy@dlpu.edu.cn) (Changyou Shao); [yangjun11@bjfu.edu.cn](mailto:yangjun11@bjfu.edu.cn) (Jun Yang); [hailincong@163.com](mailto:hailincong@163.com) (Hailin Cong)

**Note S1 Intermolecular hydrogen bonding**

Notably, the (002) peak of MXene shifts toward smaller 2θ angles with increasing TOCNF content, suggesting an increase in the average MXene interlayer spacing. Furthermore, the shift of the (002) diffraction peak from 9.3º to 7.2º and the disappearance of the (104) peak in the XRD patterns confirm the successful preparation of MXene nanosheets. Likewise, as illustrated by FTIR spectroscopy of the MXene/TOCNF composite, the peak at 3393 cm^-1^ corresponds to the O-H stretching vibration, which is characteristic of hydroxyl groups from both MXene and TOCNF. This suggests the presence of hydrogen bonding between the two components. The C=O stretching vibration peak shows a slight shift from 1730 to 1720 cm^-1^, indicating a potential interaction between the carbonyl groups of TOCNF and the functional groups on the MXene surface.

A new low-intensity peak at 1647 cm^-1^ appears, which is typically associated with bending vibrations of adsorbed water or hydrogen-bonding interactions between the MXene and TOCNF. In addition, characteristic peaks of MXene at 1165 and 525 cm^-1^, corresponding to terminal C-F and O-H groups, respectively, further confirm the presence of functional groups on the MXene surface.

**Note S2 Advantages of the CPS**

The CPS demonstrates exceptional sensitivity, a wide working range, rapid response time, and high resolution, while maintaining stability, positioning it as an advanced option for precise pressure monitoring (**Fig. S21; Table S1; Note S2**). Additionally, it excels in customizability, biodegradability, interface compliance, and gas permeability, making it ideal for flexible and sustainable applications. Mechanically, the CPS outperforms alternatives with superior toughness, bending resistance, puncture resistance, and resilience to humidity and temperature variations, ensuring reliability in harsh conditions.

**Supplementary Figures and Tables**


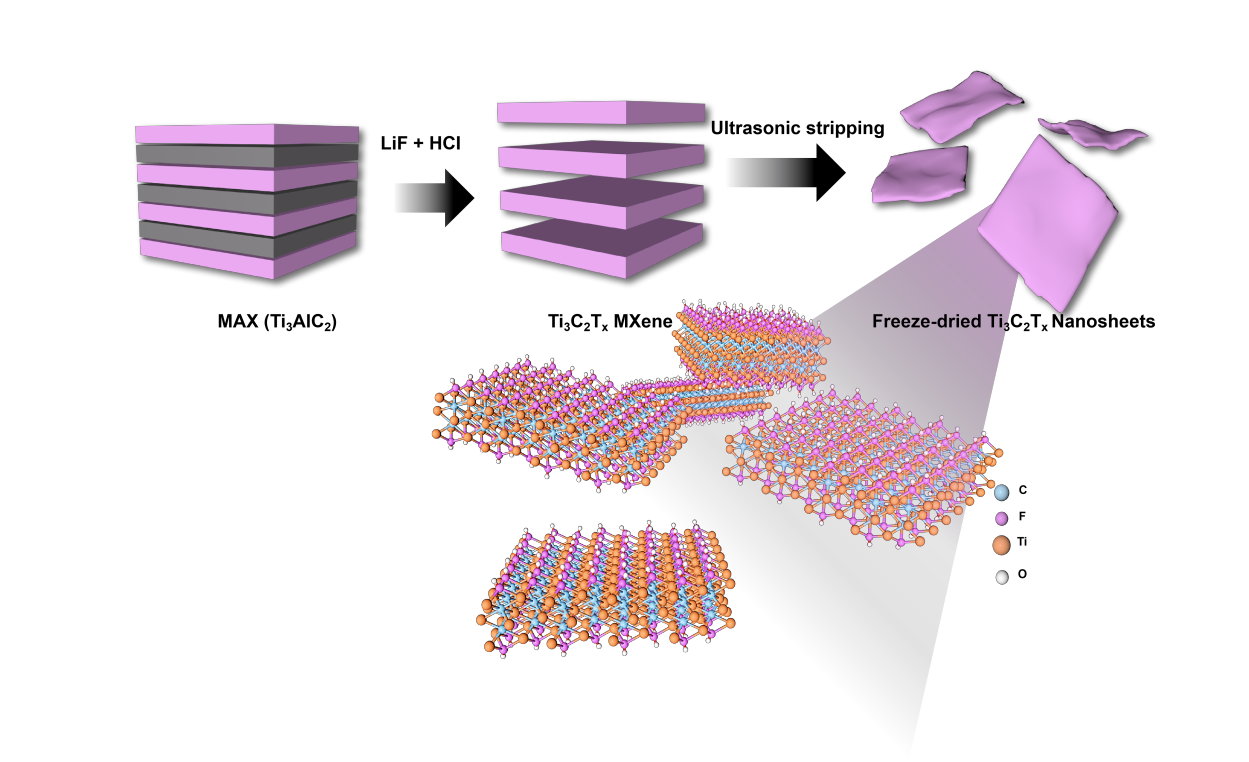


**Fig. S1** Schematic illustration of the MXene preparation process by etching Ti_3_AlC_2_ (MAX) to obtain Ti_3_C_2_T_x_ (MXene) nanosheets

**
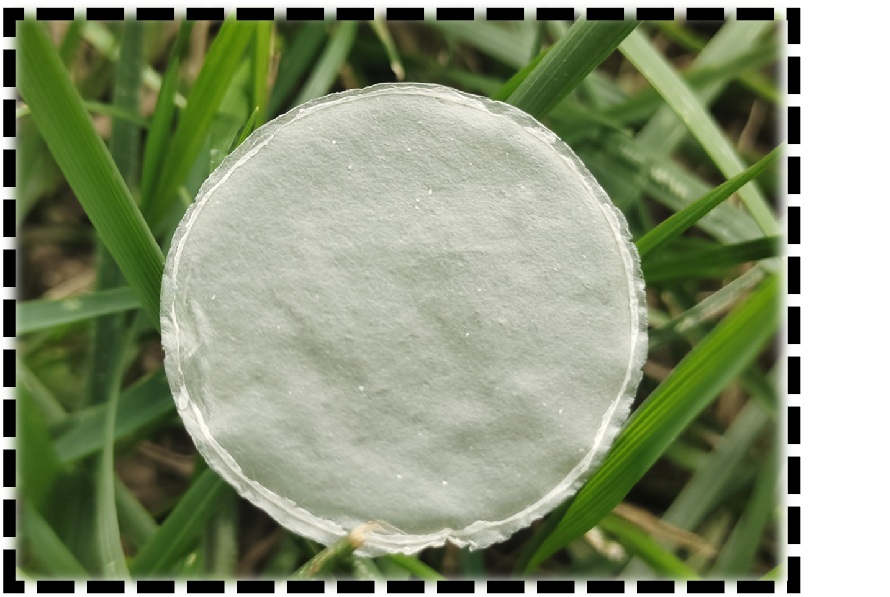
**

**Fig. S2** Picture of TOCNF paper prepared by vacuum filtration


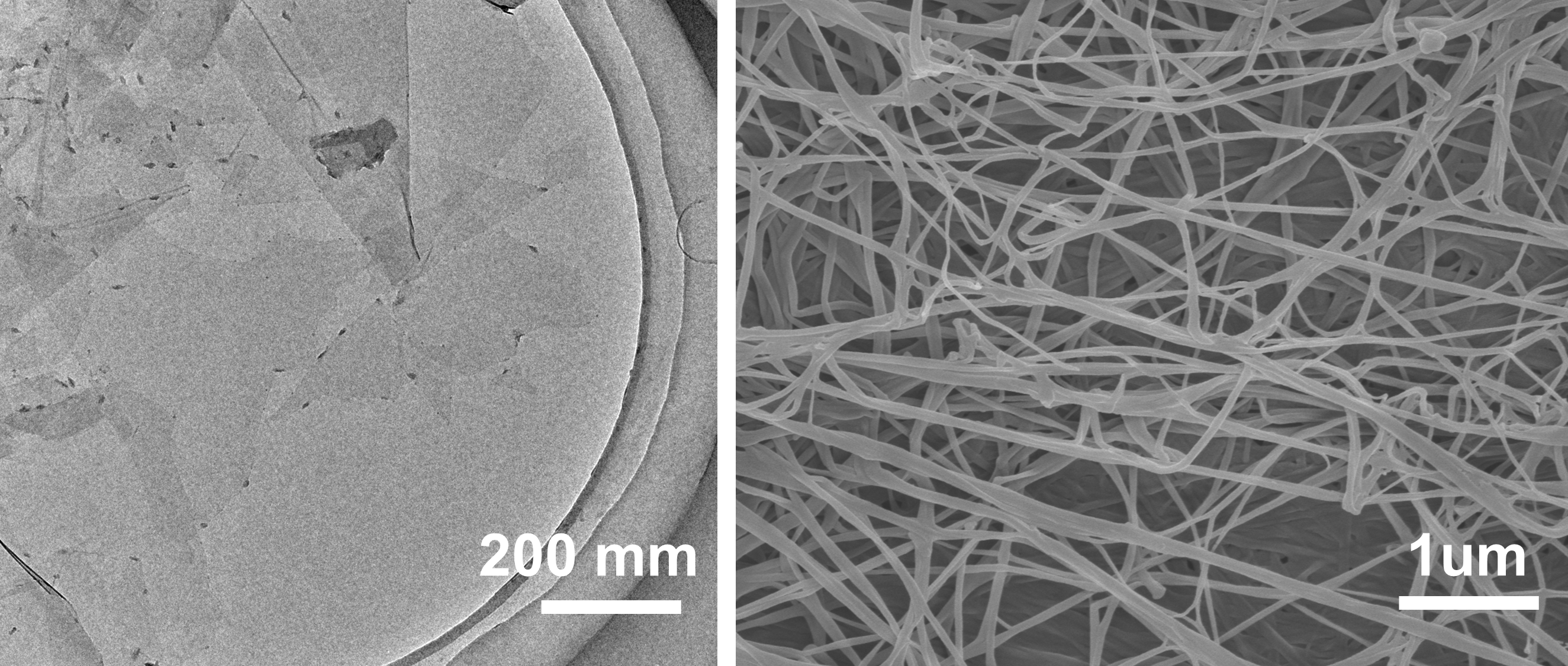


**Fig. S3** TEM image of the MXene nanosheets

**
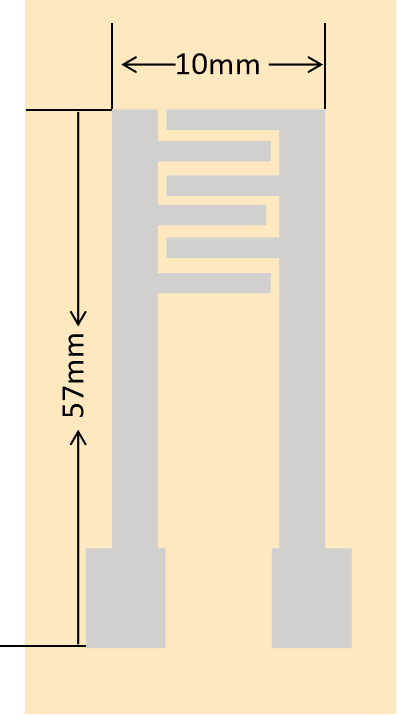
**

**Fig. S4** Scheme of Ag interdigitated electrodes


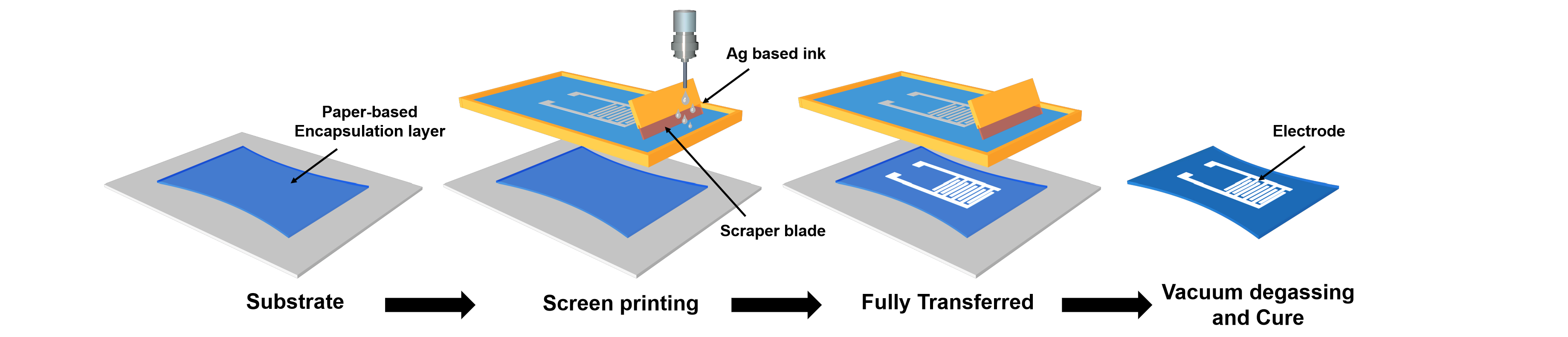


**Fig. S5** Schematic illustration of the screen-printing process for Ag interdigitated electrodes


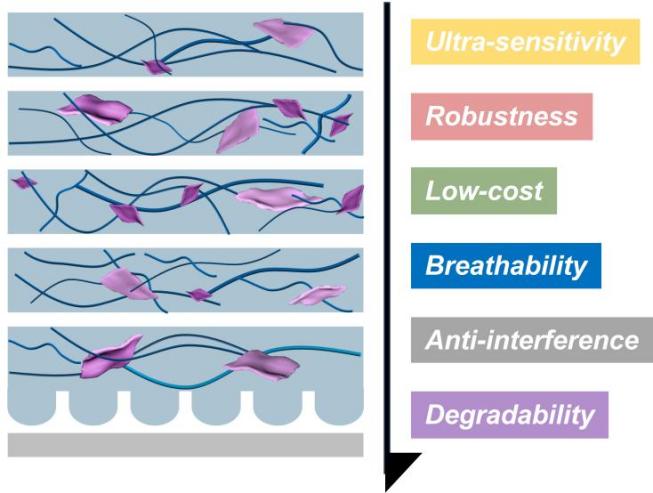


**Fig. S6** Schematic illustration of the structural feature and summary of the performance metrics


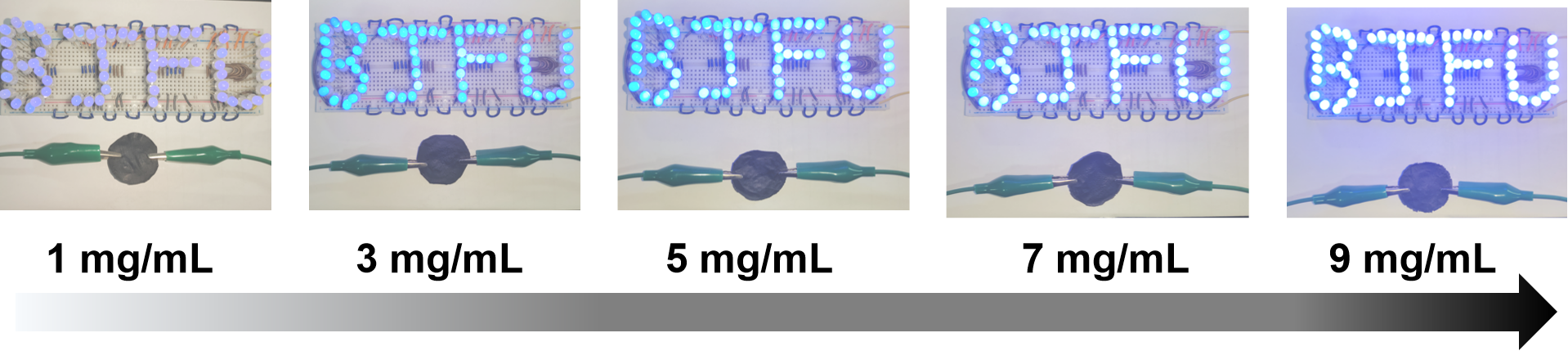


**Fig. S7** The change in brightness of a set of small bulbs with a "B, J, F, U" pattern is correlated with the CPS containing varying MXene concentrations (1-9 mg/mL)


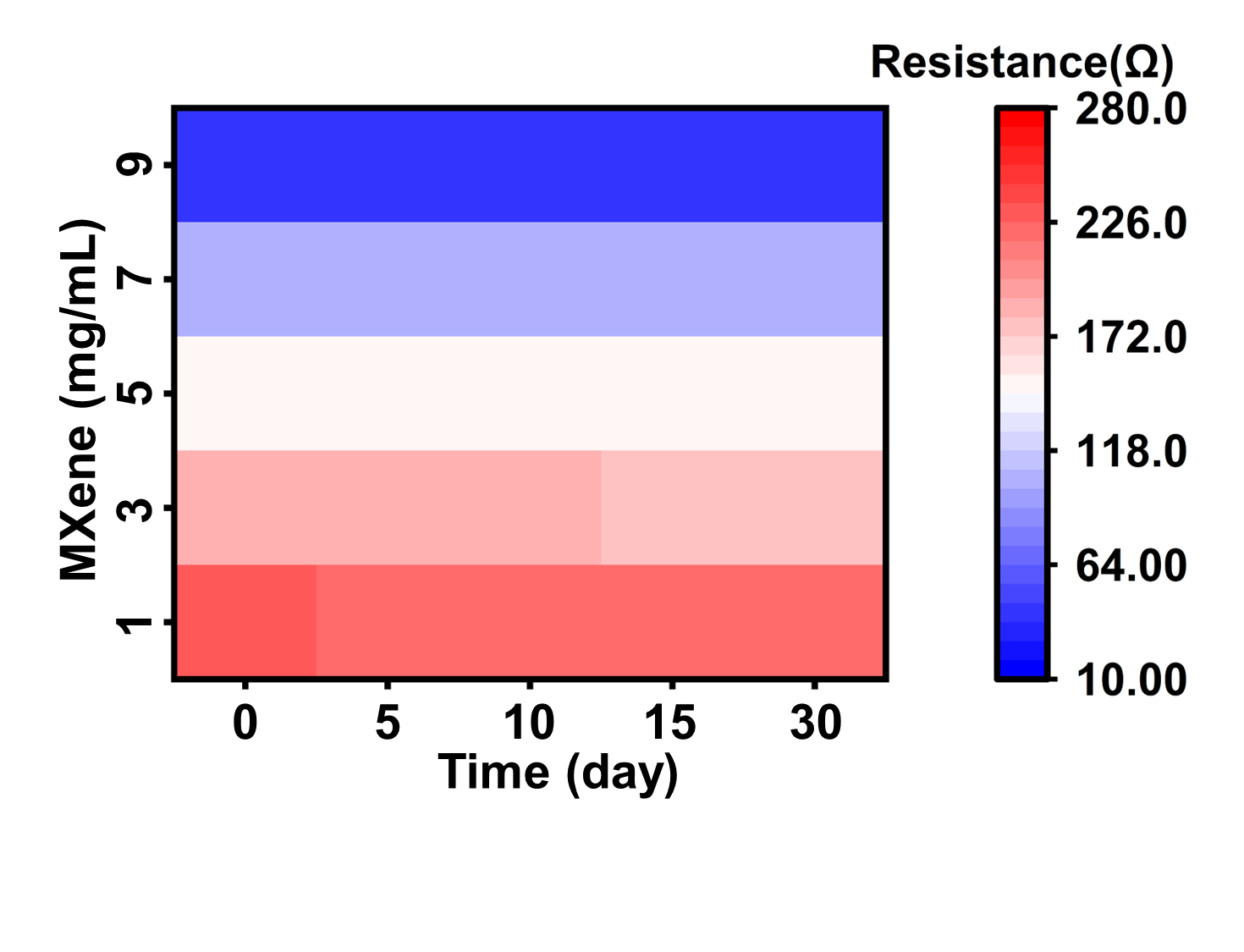


**Fig. S8** Resistance stability of MXene/TOCNF composite sensing layer

**
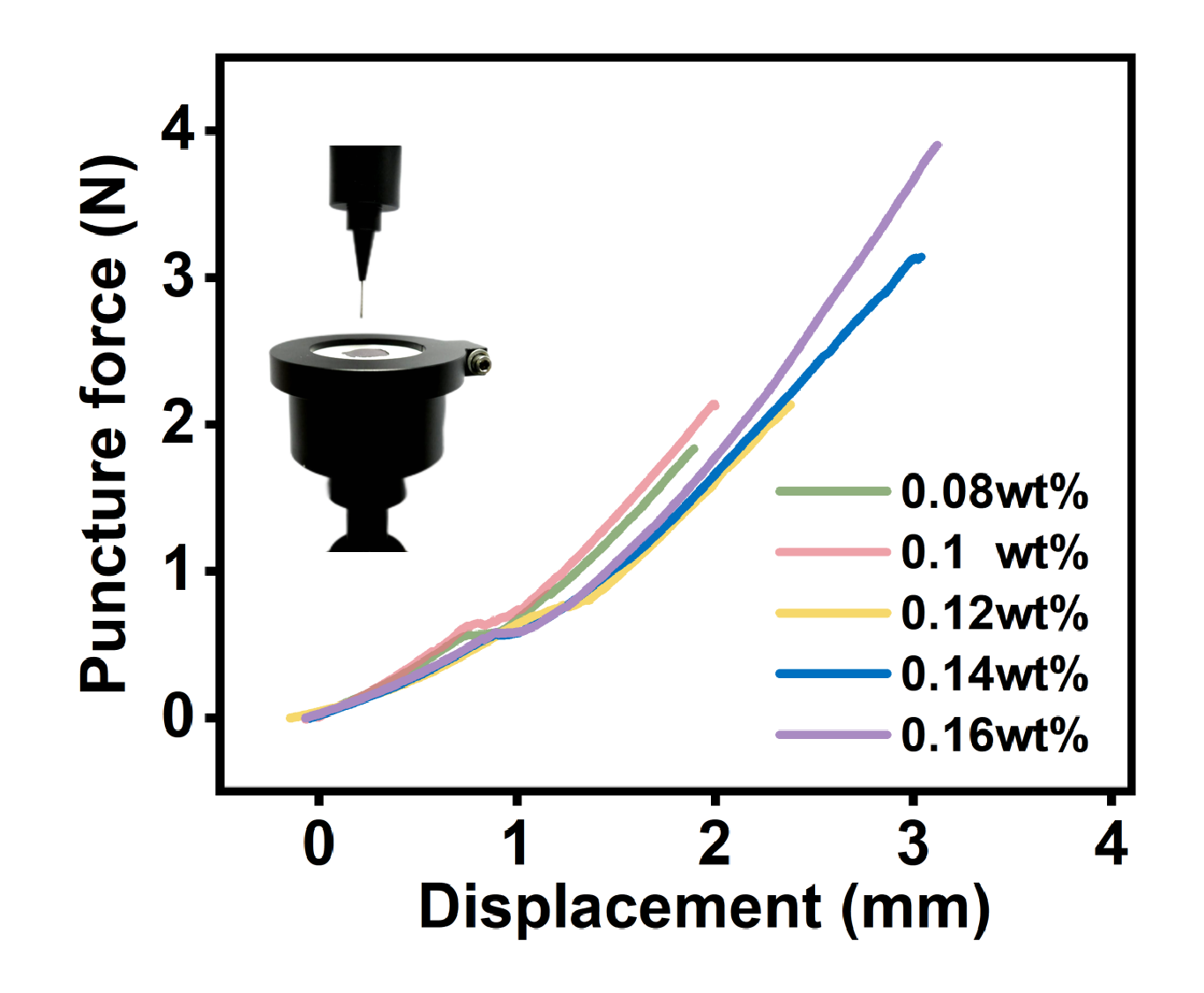
**

**Fig. S9** Puncture resistance test of CISL prepared by different concentrations of cellulose solutions (0.08 wt% -0.16 wt%)


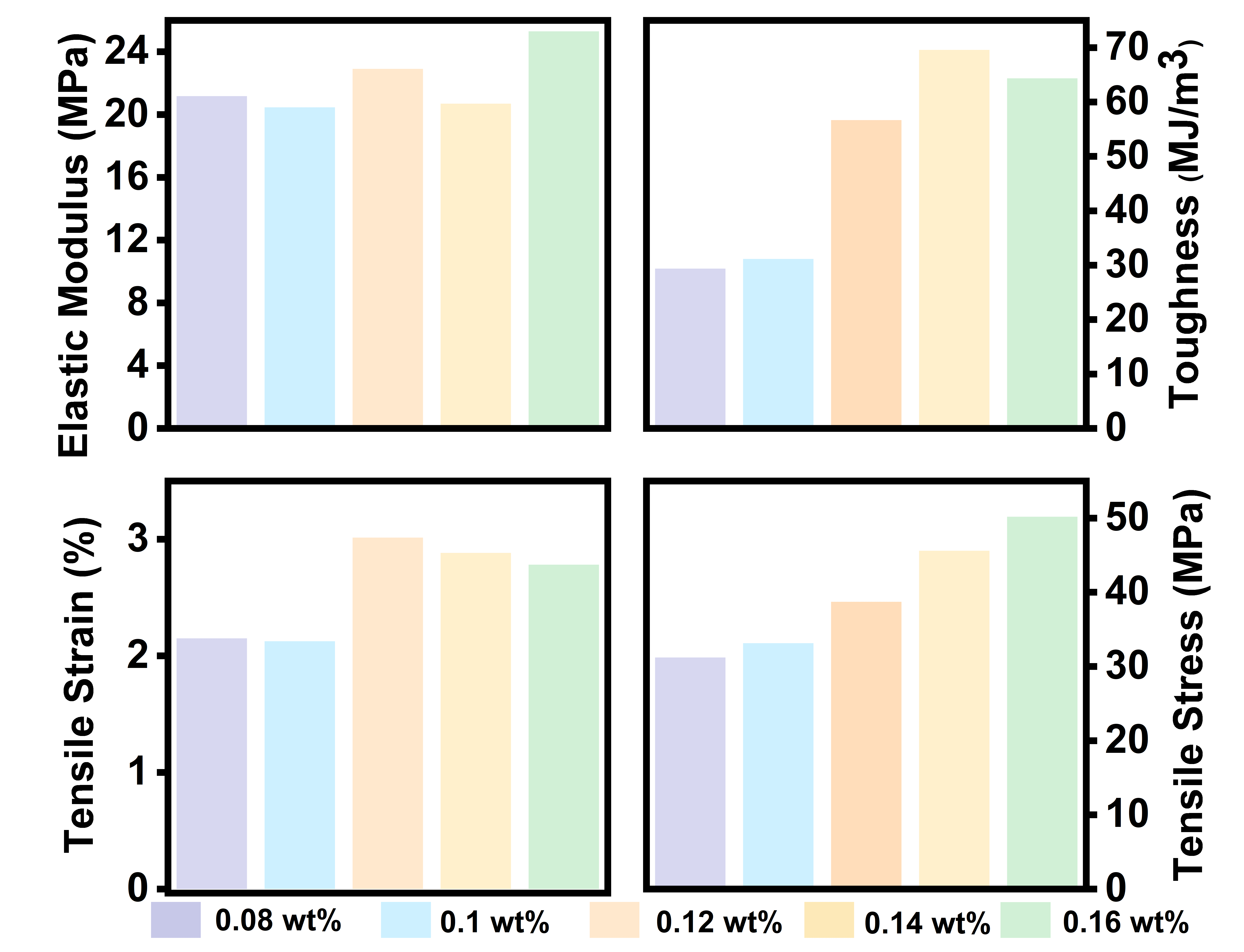


**Fig. S10** Detailed mechanical data of CISL including: elastic modulus, toughness, stress and strain


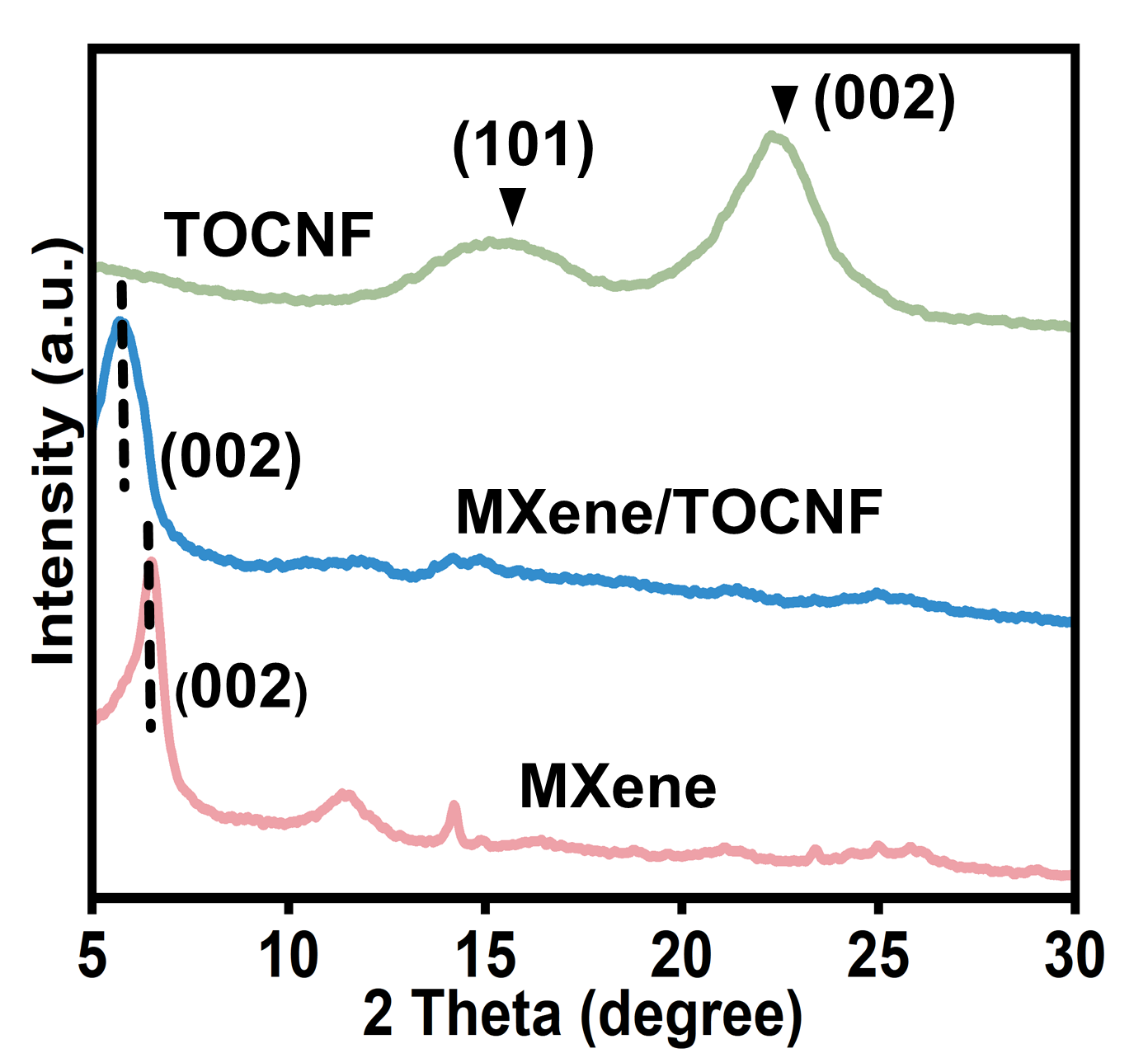


**Fig. S11** XRD patterns of TOCNF, MXene/TOCNF and MXene


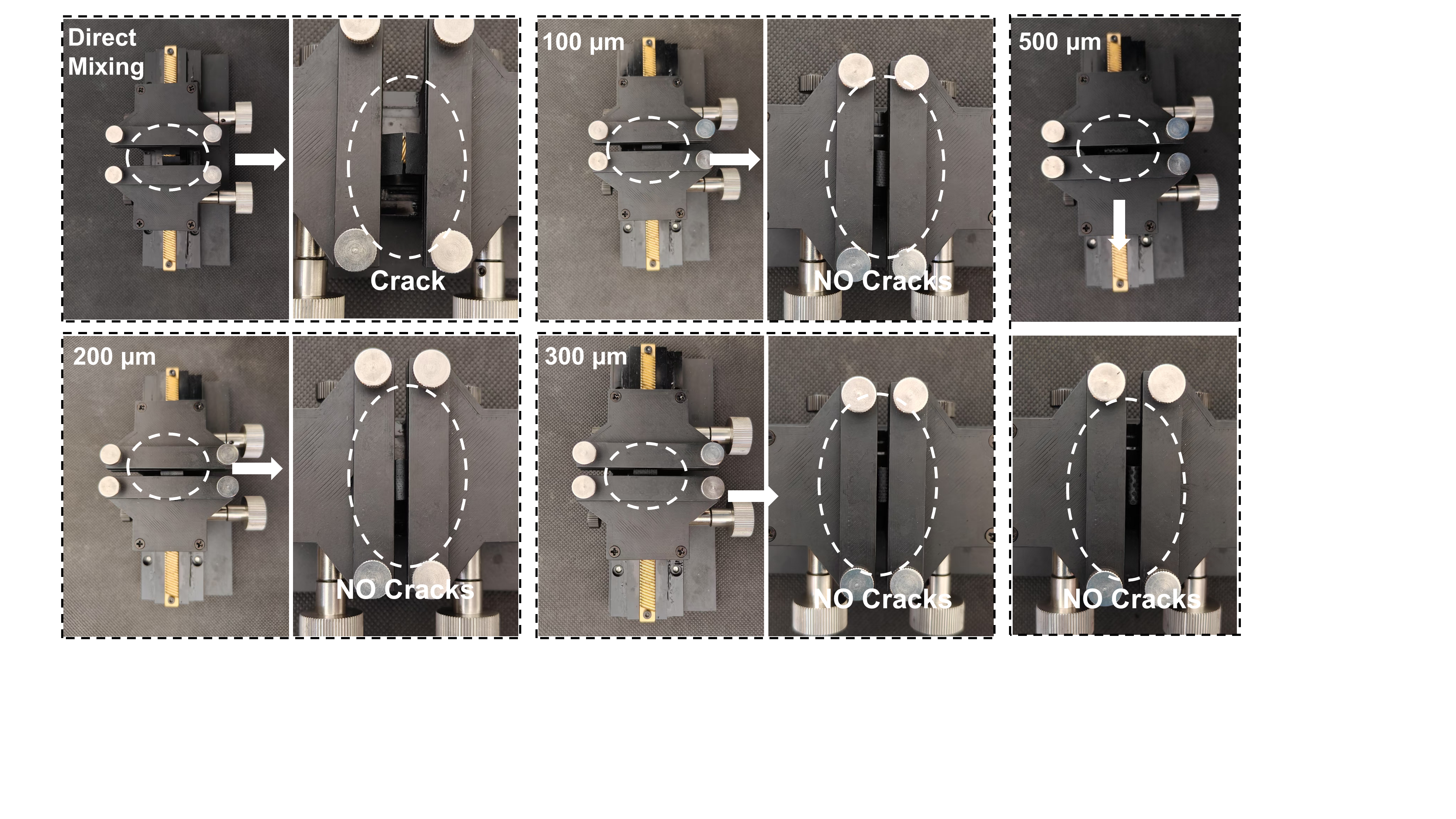


**Fig. S12** Bending results of MXene/cellulose nanofibril layers: cracks appear with direct mixing, while vacuum-assisted self-assembly prevents cracking


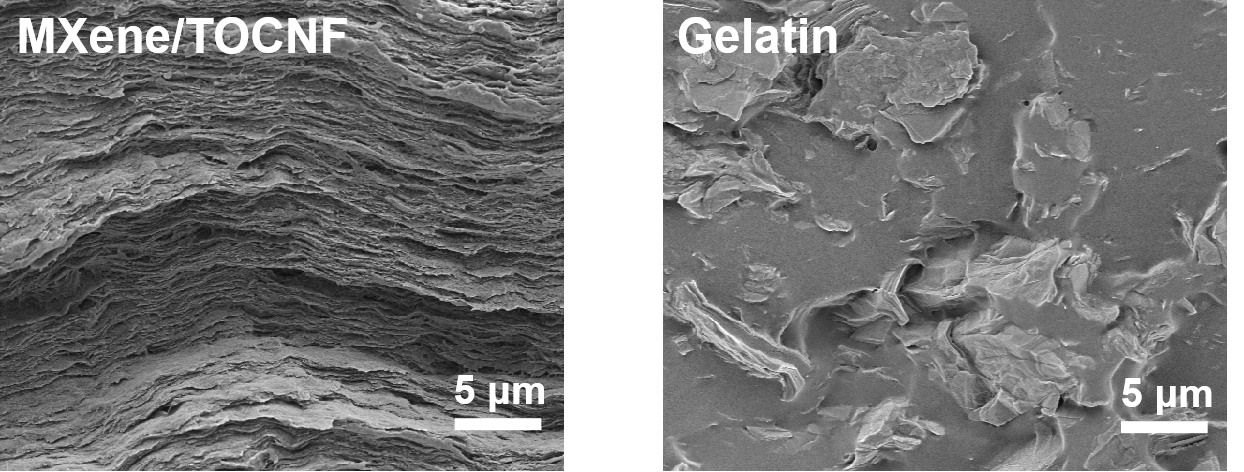


**Fig. S13** SEM image of the MXene/TOCNF and gelatin


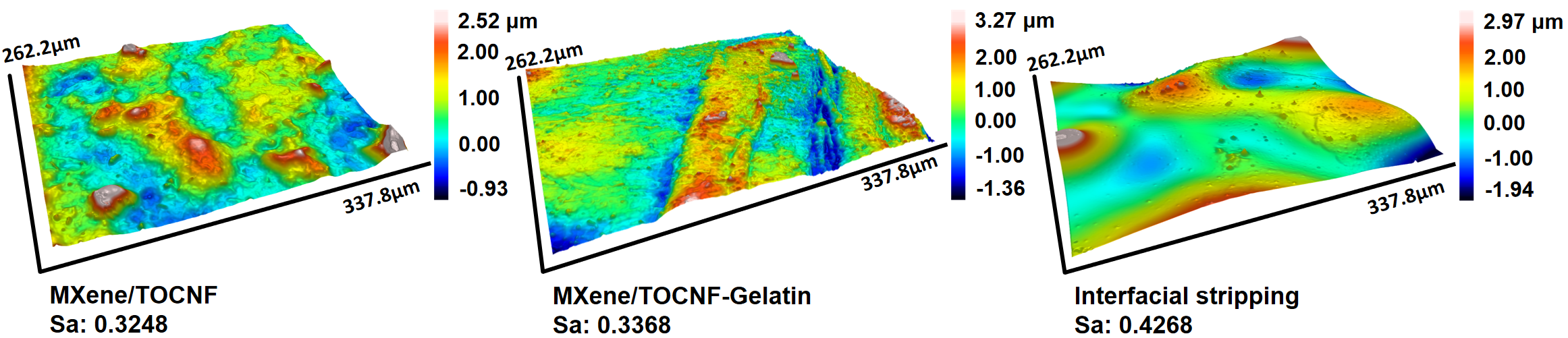


**Fig. S14** The 3D confocal laser scanning microscope (CLSM) images for the surface microstructure variation

**
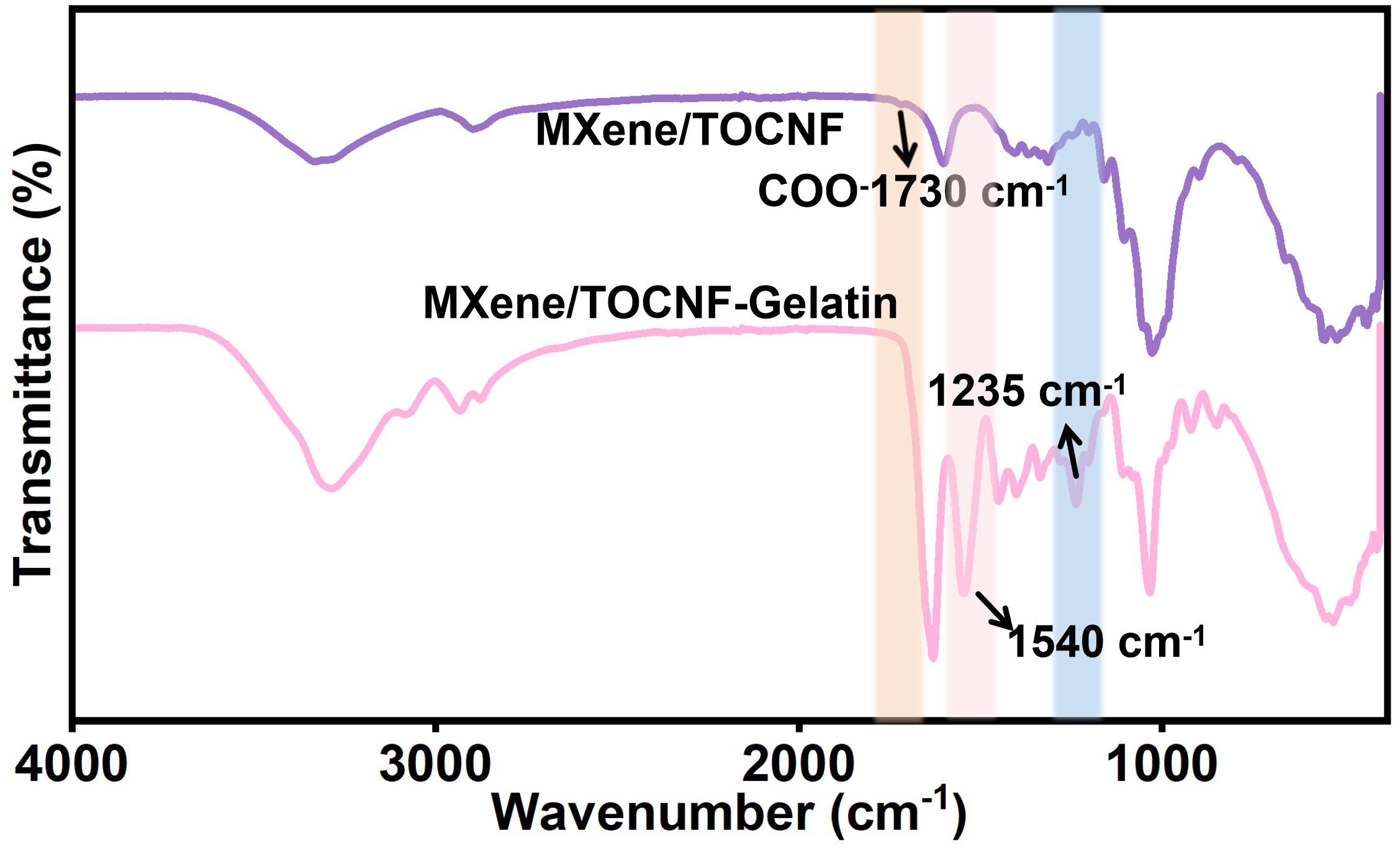
**

**Fig. S15** FTIR spectra of the MXene/TOCNF, and MXene/TOCNF-Gelatin, respectively


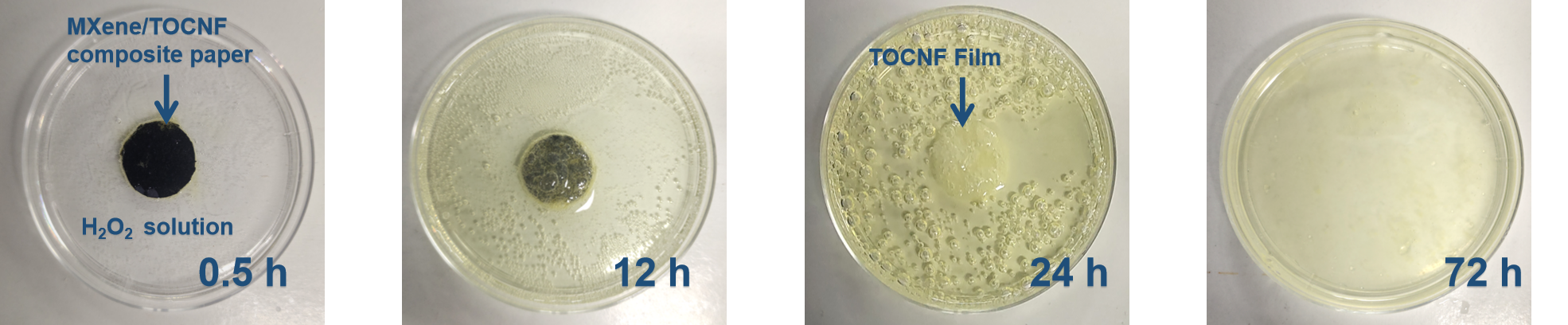


**Fig. S16** The CISL degradation and nanofibril disintegration occurred in a 4 wt % H_2_O_2_ solution at room temperature after 72h


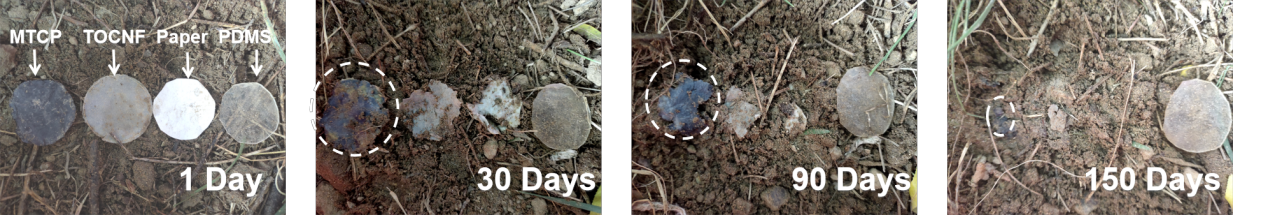


**Fig. S17** The images of the degradation process for TOCNF paper, paper, PDMS and the CISL over 150 Days


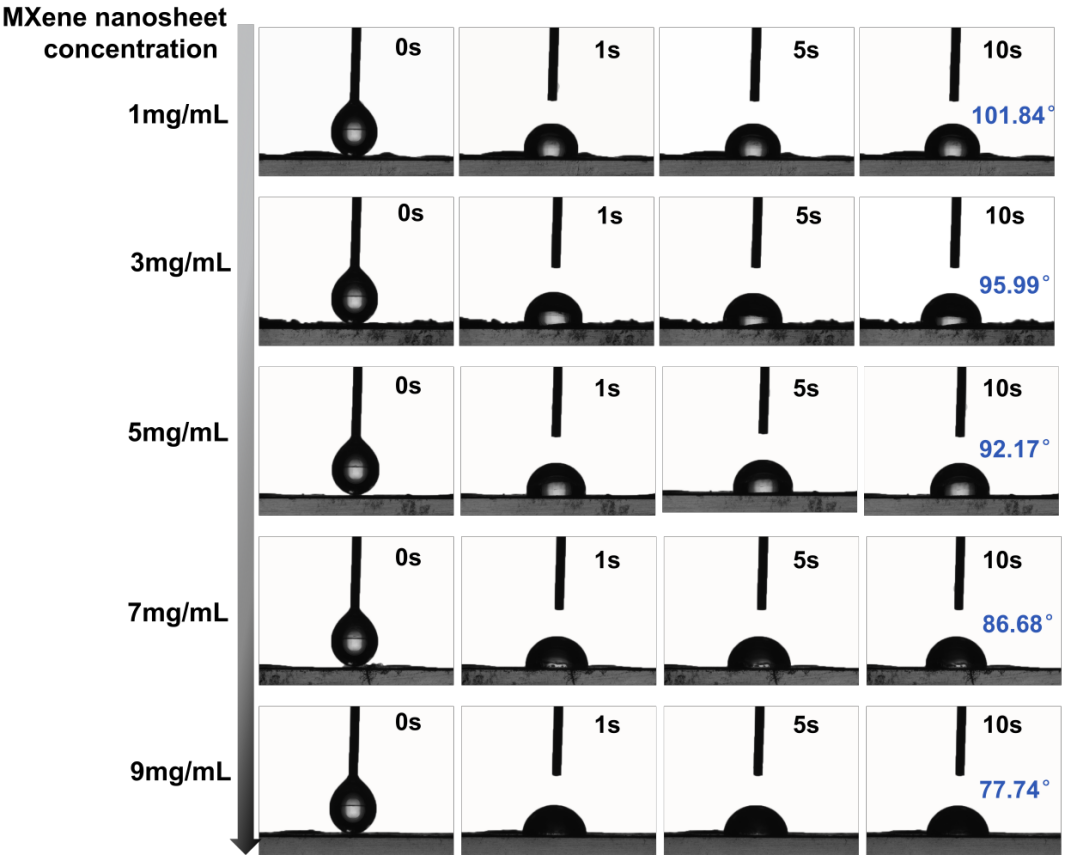


**Fig. S18** The hydrophobicity features of CISL associated with increasing of MXene nanosheets content (1-9 mg/mL) in 10 consecutive seconds

**
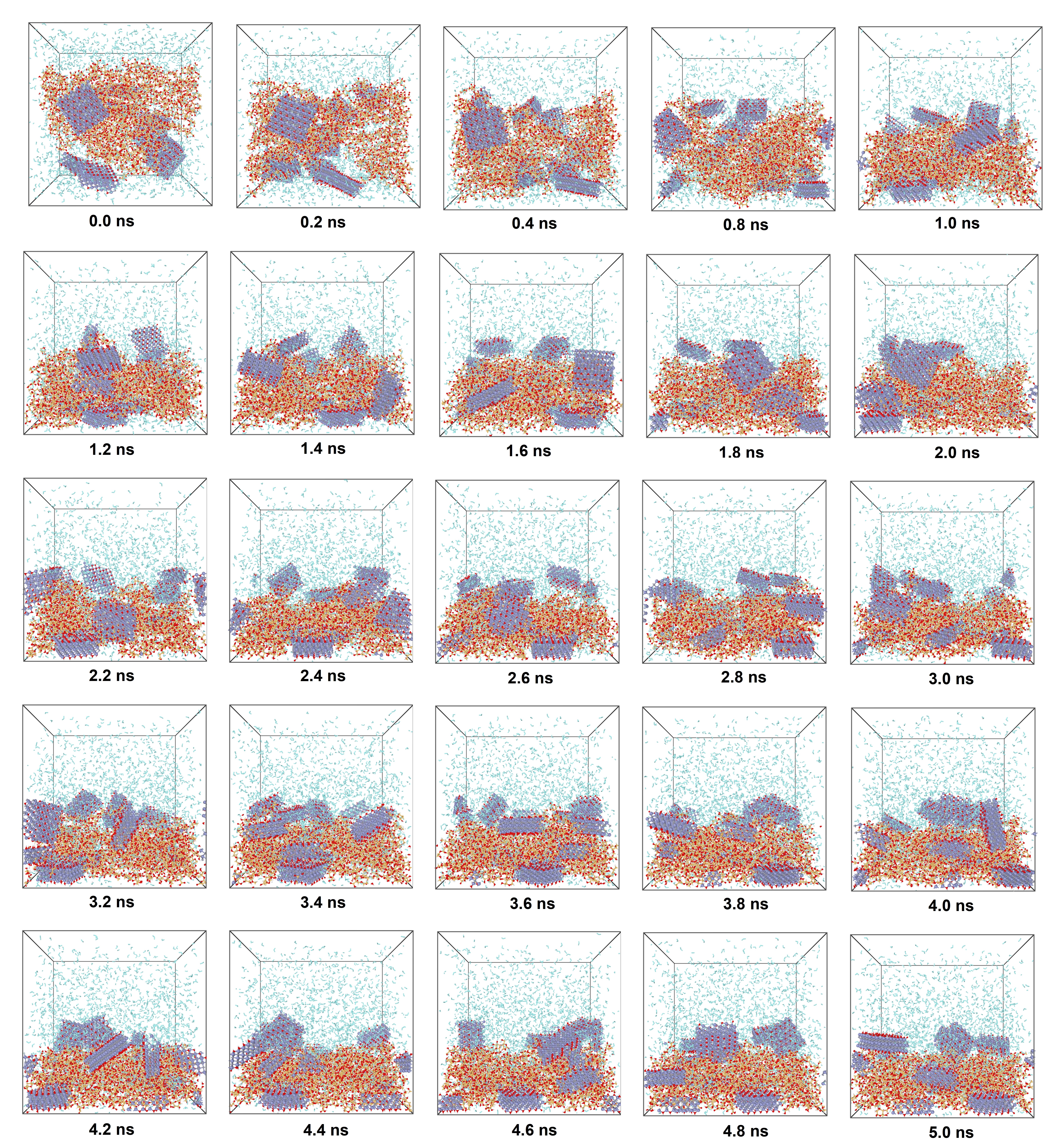
**

**Fig. S19** The simulation configurations under shear force fields from 0.0 to 5.0 ns are presented

**Fig. S20** Response time and recover time of CPS with different sensing layer

**Fig. S21** Pressure response stability with different puncture cycles (1, 10, 20, and 50)


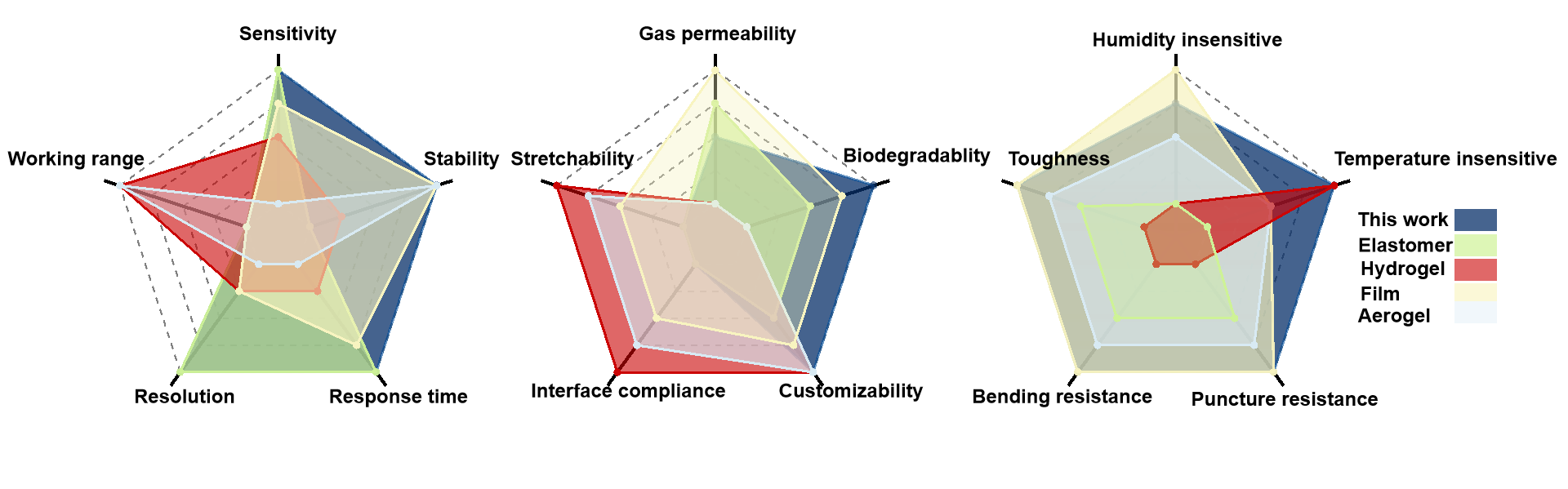


**Fig. S22** Radar plots comparing sensors based on the aforementioned materials [S5-S10]


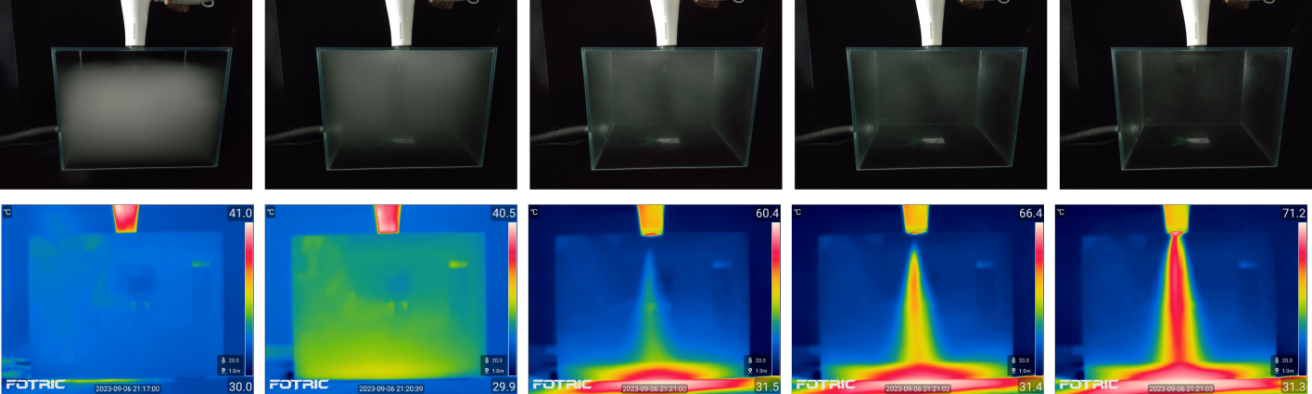


**Fig. S23** The images of anti-crosstalk test procedure in fog chamber and the corresponding thermal photos


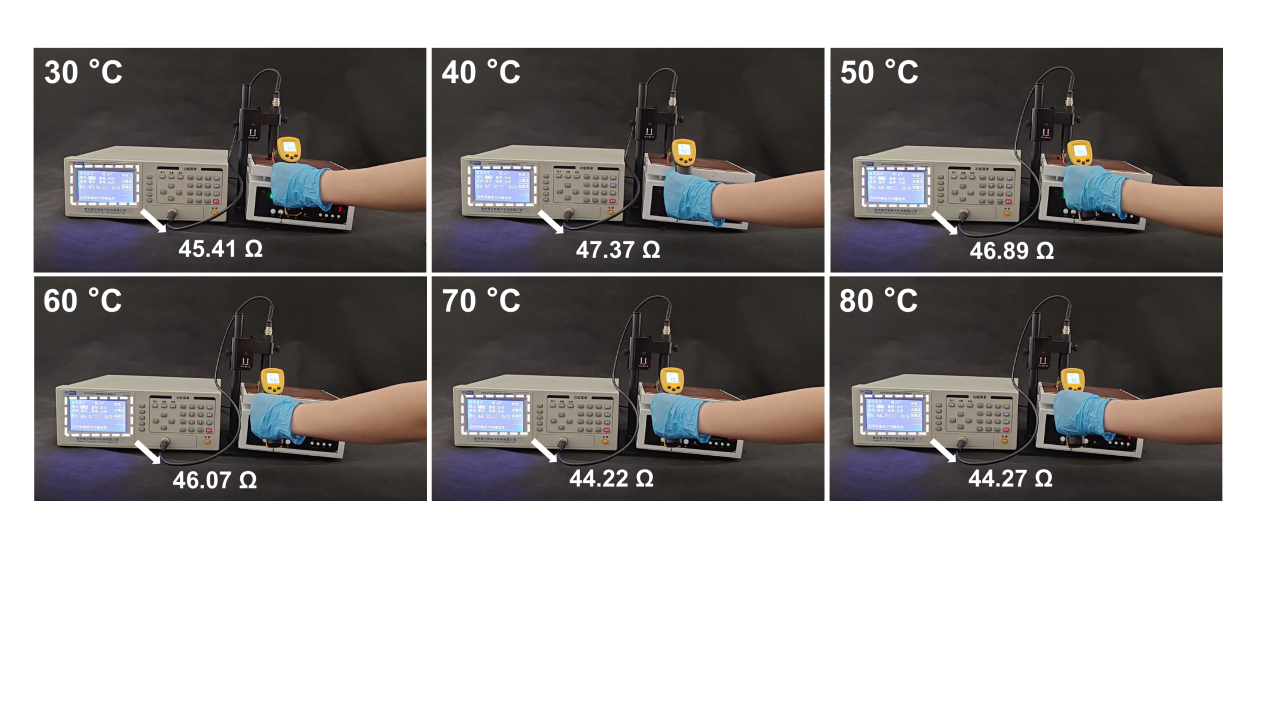


**Fig. S24** Schematic illustration of the resistance stability mechanism of the sensing layer under varying temperatures (30-80 ℃)


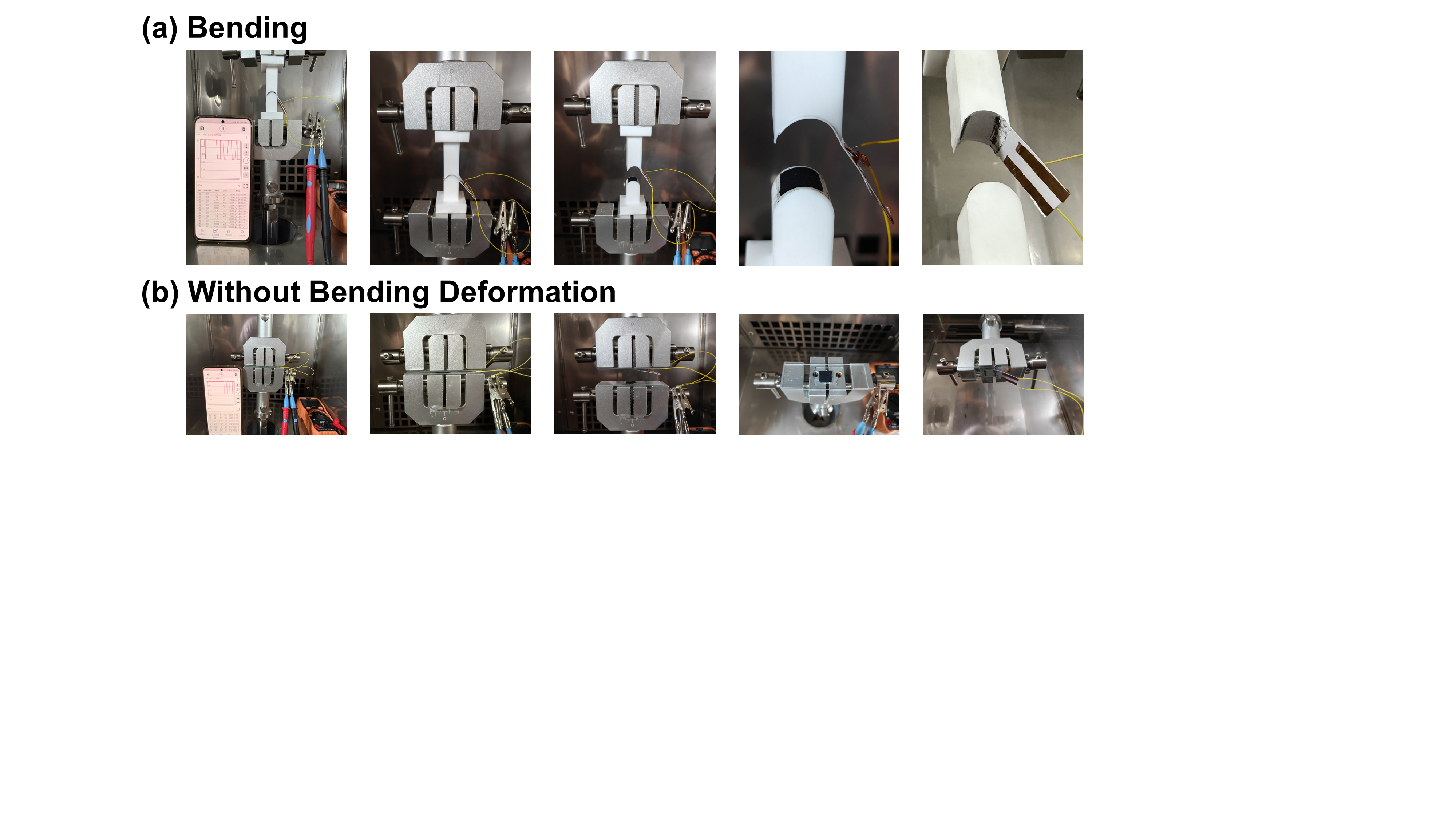


**Fig. S25** Real-time output stability of the packaged piezoresistive sensor under bending and without bending deformation conditions


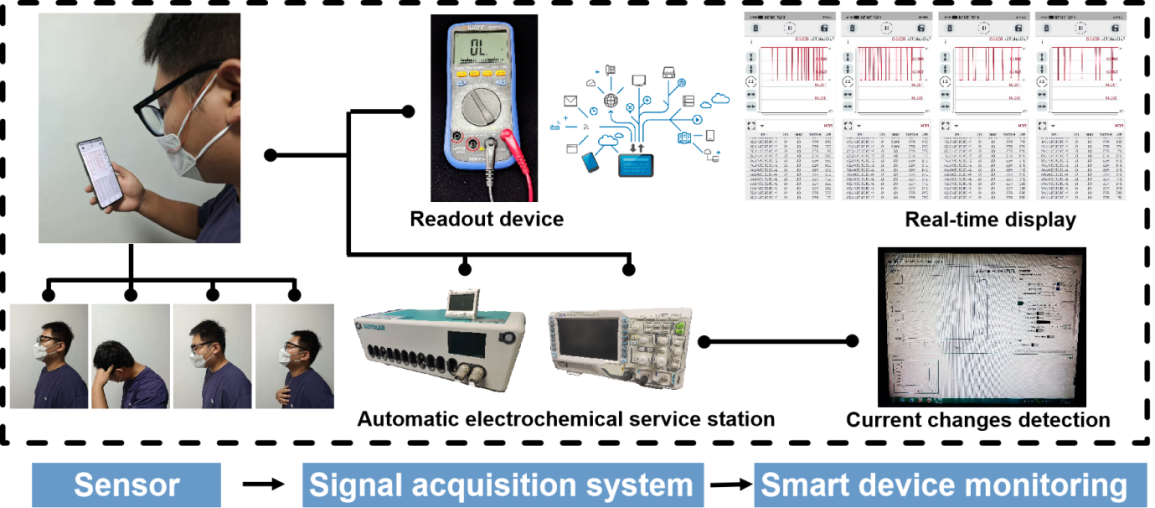


**Fig. S26** The flowchart of respiratory rate monitoring


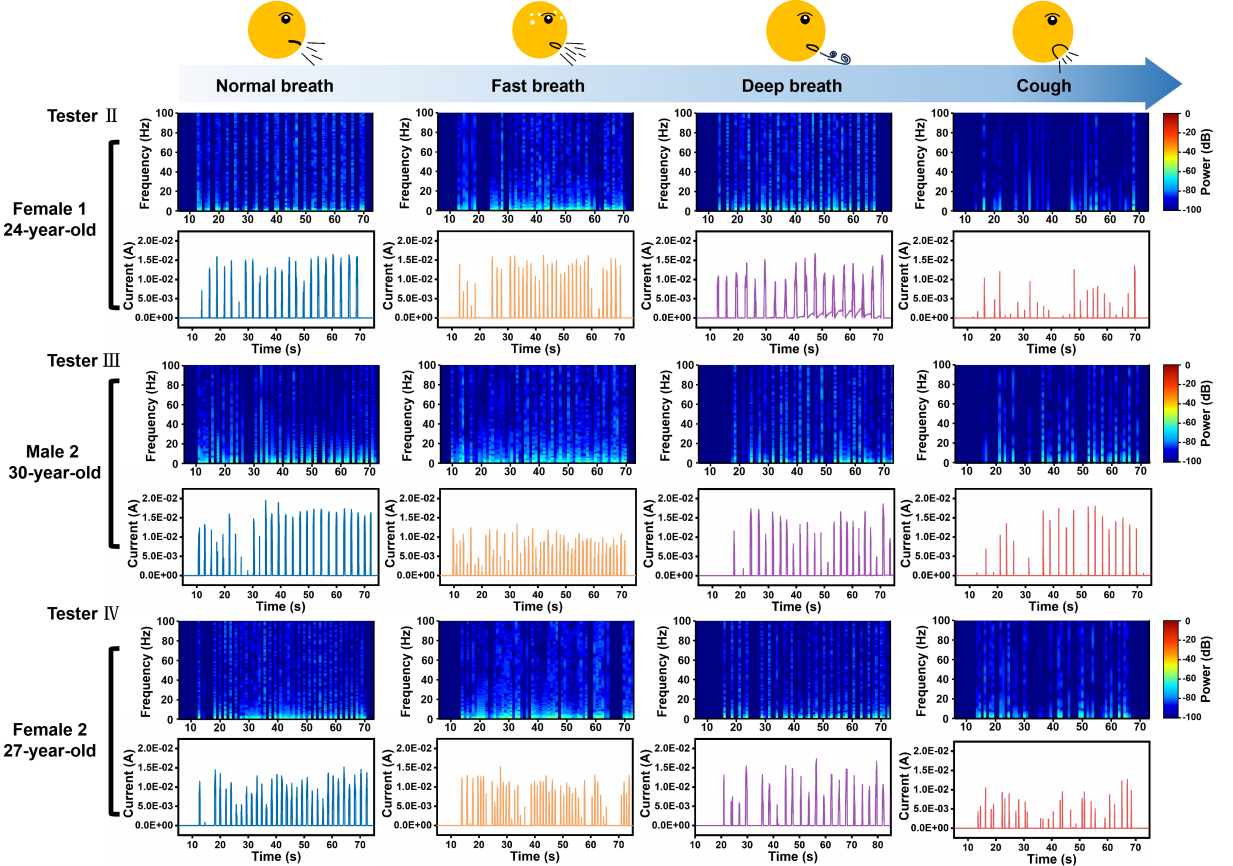


**Fig. S27 Validation of respiration.** The stable and accurate breathing signals recording and the corresponding time-frequency domain analysis in terms of normal breath, fast breath, deep breath, and cough conditions.

**Table S1** Comparison of the collection features and competitive piezoresistive performances of our cellulose-based piezoresistive sensor in this work with previously reported piezoresistive sensors.


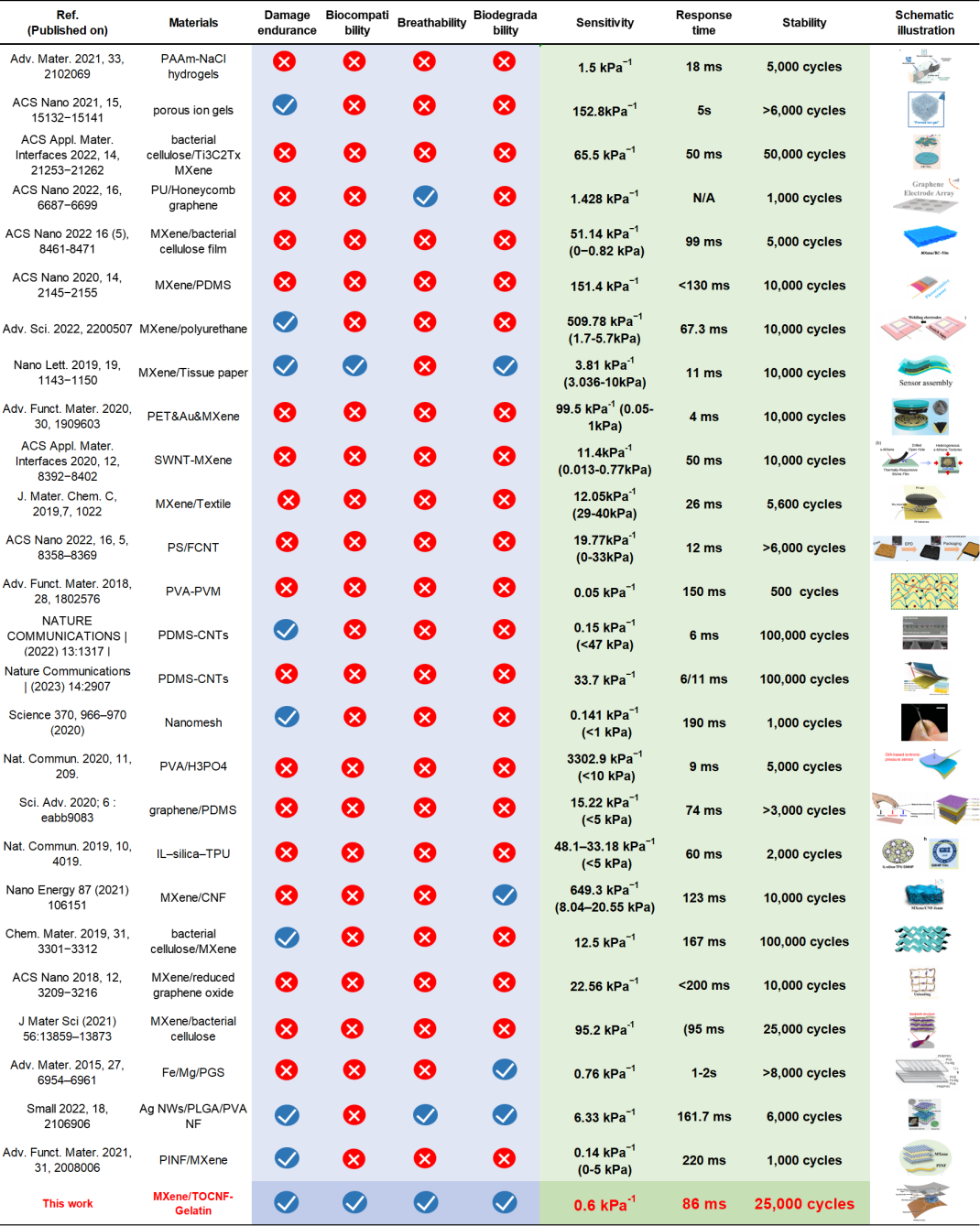


**Supplementary References**

1. A.P. Thompson, H.M. Aktulga, R. Berger, D.S. Bolintineanu, W.M. Brown et al., LAMMPS - a flexible simulation tool for particle-based materials modeling at the atomic, meso, and continuum scales. Comput. Phys. Commun. **271**, 108171 (2022). <https://doi.org/10.1016/j.cpc.2021.108171>
2. G.A. Kaminski, R.A. Friesner, J. Tirado-Rives, W.L. Jorgensen, Evaluation and reparametrization of the OPLS-AA force field for proteins *via* comparison with accurate quantum chemical calculations on peptides. J. Phys. Chem. B **105**(28), 6474–6487 (2001). <https://doi.org/10.1021/jp003919d>
3. A.K. Rappe, C.J. Casewit, K.S. Colwell, W.A. III Goddard, W.M. Skiff, UFF, a full periodic table force field for molecular mechanics and molecular dynamics simulations. J. Am. Chem. Soc. **114**(25), 10024–10035 (1992). <https://doi.org/10.1021/ja00051a040>
4. H.J.C. Berendsen, J.R. Grigera, T.P. Straatsma, The missing term in effective pair potentials. J. Phys. Chem. **91**(24), 6269–6271 (1987). <https://doi.org/10.1021/j100308a038>
5. X. Wei, H. Li, W. Yue, S. Gao, Z. Chen et al., A high-accuracy, real-time, intelligent material perception system with a machine-learning-motivated pressure-sensitive electronic skin. Matter **5**(5), 1481–1501 (2022). <https://doi.org/10.1016/j.matt.2022.02.016>
6. G. Song, Y. Zhan, Y. Hu, J. Rao, N. Li et al., Paper-mill waste reinforced nanofluidic membrane as high-performance osmotic energy generators. Adv. Funct. Mater. **33**(26), 2214044 (2023). <https://doi.org/10.1002/adfm.202214044>
7. G. Ge, Y. Zhang, J. Shao, W. Wang, W. Si et al., Stretchable, transparent, and self-patterned hydrogel-based pressure sensor for human motions detection. Adv. Funct. Mater. **28**(32), 1802576 (2018). <https://doi.org/10.1002/adfm.201802576>
8. Y. Shin, Y.W. Kim, H.J. Kang, J.H. Lee, J.E. Byun et al., Stretchable and skin-mountable temperature sensor array using reduction-controlled graphene oxide for dermatological thermography. Nano Lett. **23**(11), 5391–5398 (2023). <https://doi.org/10.1021/acs.nanolett.2c04752>
9. L.-Q. Tao, K.-N. Zhang, H. Tian, Y. Liu, D.-Y. Wang et al., Graphene-paper pressure sensor for detecting human motions. ACS Nano **11**(9), 8790–8795 (2017). <https://doi.org/10.1021/acsnano.7b02826>
10. W.-T. Cao, F.-F. Chen, Y.-J. Zhu, Y.-G. Zhang, Y.-Y. Jiang et al., Binary strengthening and toughening of MXene/cellulose nanofiber composite paper with nacre-inspired structure and superior electromagnetic interference shielding properties. ACS Nano **12**(5), 4583–4593 (2018). <https://doi.org/10.1021/acsnano.8b00997>
